# NrnA is a 5′-3′ exonuclease that processes short RNA substrates *in vivo* and *in vitro*

**DOI:** 10.1093/nar/gkac1091

**Published:** 2022-12-07

**Authors:** Cordelia A Weiss, Tanner M Myers, Chih Hao Wu, Conor Jenkins, Holger Sondermann, Vincent T Lee, Wade C Winkler

**Affiliations:** Department of Cell Biology and Molecular Genetics, University of Maryland, College Park, MD 20742, USA; Department of Chemistry and Biochemistry, University of Maryland, College Park, MD 20742, USA; Department of Cell Biology and Molecular Genetics, University of Maryland, College Park, MD 20742, USA; Department of Chemistry and Biochemistry, University of Maryland, College Park, MD 20742, USA; CSSB – Centre for Structural Systems Biology, Deutsches Elektronen-Synchrotron (DESY), 22607 Hamburg, Germany; Christian-Albrechts-Universität, 24118 Kiel, Germany; Department of Cell Biology and Molecular Genetics, University of Maryland, College Park, MD 20742, USA; Department of Cell Biology and Molecular Genetics, University of Maryland, College Park, MD 20742, USA; Department of Chemistry and Biochemistry, University of Maryland, College Park, MD 20742, USA

## Abstract

Bacterial RNases process RNAs until only short oligomers (2–5 nucleotides) remain, which are then processed by one or more specialized enzymes until only nucleoside monophosphates remain. Oligoribonuclease (Orn) is an essential enzyme that acts in this capacity. However, many bacteria do not encode for Orn and instead encode for NanoRNase A (NrnA). Yet, the catalytic mechanism, cellular roles and physiologically relevant substrates have not been fully resolved for NrnA proteins. We herein utilized a common set of reaction assays to directly compare substrate preferences exhibited by NrnA-like proteins from *Bacillus subtilis*, *Enterococcus faecalis*, *Streptococcus pyogenes* and *Mycobacterium tuberculosis*. While the *M. tuberculosis* protein specifically cleaved cyclic di-adenosine monophosphate, the *B. subtilis, E. faecalis* and *S. pyogenes* NrnA-like proteins uniformly exhibited striking preference for short RNAs between 2–4 nucleotides in length, all of which were processed from their 5′ terminus. Correspondingly, deletion of *B. subtilis nrnA* led to accumulation of RNAs between 2 and 4 nucleotides in length in cellular extracts. Together, these data suggest that many Firmicutes NrnA-like proteins are likely to resemble *B. subtilis* NrnA to act as a housekeeping enzyme for processing of RNAs between 2 and 4 nucleotides in length.

## INTRODUCTION

The general cycle of RNA metabolism involves polymerization of NTPs by DNA-dependent RNA polymerase, followed by fragmentation and hydrolysis of cellular RNAs by ribonucleolytic enzymes (RNases). The process of RNA degradation is essential, as it is required for replenishing NTP pools; therefore, RNA degradation pathways profoundly affect gene expression, environmental adaptation, and cellular viability. Moreover, defects in RNA degradation may lead to aberrant NTP pools that interfere with nucleotide signaling pathways. While the general principles of RNA processing and decay are conserved among bacteria, the individual strategies and classes of RNases differ from microbe to microbe. As such, there is no universally conserved set of RNases that all bacteria rely upon for mRNA turnover (1). Indeed, of the 40 RNases that have been identified for *Escherichia coli* and *Bacillus subtilis*, only a subset is shared between the two microorganisms (2). For example, while bulk *E. coli* mRNA degradation is typically initiated by endoribonuclease E (RNase E), which is essential in *E. coli* and other Gammaproteobacteria, RNase E is conspicuously absent in the genomes of *B. subtilis* and many Firmicutes. Instead, *B. subtilis* employs endoribonuclease Y (RNase Y) as a key enzyme to initiate global mRNA turnover (3). The action of endonucleolytic cleavage results in two mRNA decay products that are no longer protected at the 3′ (upstream product) or 5′ (downstream product) terminus, which can then be subject to degradation by exoribonucleases. Generally, the exoribonucleases are thought to act processively, cleaving in the 3′-5′ direction. However, for some bacteria, RNAs are also processed from the 5′ terminus by RNase J1 or RNase AM (YciV), which are the only 5′-3′ exoribonucleases to be discovered in bacteria to date (4,5). The combined action of RNase enzymes leads to processing of mRNAs until only short (< 5 nucleotides) RNA fragments remain. Then, a separate category of RNase enzymes is required for processing of these short RNAs.

In *E. coli* and other bacteria, the enzyme Oligoribonuclease (Orn) performs the crucial terminal step of RNA degradation. Originally discovered in the 1960s, it was argued that Orn is a 3′-5′ exoribonuclease that specifically cleaves short oligoribonucleotides from between 2 and 5 nucleotides in length (6–10). Interestingly, Orn is essential for viability in several species where it has been tested; however, the molecular basis of Orn's essentiality is not yet known (9). This essentiality makes Orn unique among all other known 3′-5′ exoribonucleases, which have been shown in some instances to be functionally redundant. Yet, a recent biochemical reassessment of Orn revealed that it binds and cleaves dinucleotides with greater specificity than other short RNAs (11). Similarly, an X-ray crystallographic analysis of RNA-bound Orn revealed a substrate binding pocket that appears to be remarkably restricted to dinucleotides. Based on these combined data, it was proposed that Orn might function most of the time as a ‘diribonuclease’, an enzyme that preferentially acts upon diribonucleotides rather than longer RNA substrates (11). This discovery suggests that processing of very small RNA species may occur through discrete steps and that cleavage of dinucleotides might represent a particularly crucial bottleneck in RNA degradation. Yet not all bacteria encode for Orn. In fact, the phylogenetic distribution of *orn* is limited to Proteobacteria and Actinobacteria, as well as Eukaryotes. Therefore, it is important to discover what alternative strategies bacteria utilize for degradation of diribonucleotides and other short RNAs (12–15).

A few candidate proteins have been discovered to have overlapping roles with Orn. These proteins were identified based on their ability to complement the growth defect elicited by a conditional *E. coli orn* mutant (14–16). Based on this complementation phenotype, it was suggested that these proteins participate in the terminal steps of RNA degradation, akin to Orn. Two of these RNases are encoded by *B. subtilis*: NanoRNase A (NrnA*_Bs_*) and NanoRNase B (NrnB*_Bs_*). Since their discovery, it has been largely assumed that NrnA*_Bs_* and NrnB*_Bs_* are likely to behave in a redundant manner to cleave oligoribonucleotides. While Orn's active site includes a signature DEDD(h) amino acid motif, NrnA*_Bs_* and NrnB*_Bs_* are both members of the DHH superfamily family of proteins (17). This superfamily consists of enzymes that exhibit DNA or RNA phosphodiesterase activity that together encompass a broad range of substrate specificities. Proteins in this superfamily display a conserved N-terminal domain, responsible for their inclusion in the DHH superfamily (Pfam PF01368), while sequences of the C-terminal domain have been found to vary among family members. For example, a subset of proteins in the DHH superfamily contain an associated C-terminal DHHA1 (DHH-associated domain 1, Pfam PF02272) domain. This DHHA1 subfamily includes proteins of diverse functions, such as NrnA proteins, cyclic-di-adenosine monophosphate (c-di-AMP) phosphodiesterase GdpP, alanyl-tRNA synthetase (AlaRS), and RecJ (18,19). RecJ is a 5′-3′ exonuclease that processes single-stranded DNA (ssDNA) and is involved in homologous recombination and DNA repair (20). Therefore, not all proteins with DHH and DHHA1 motifs display the same nuclease activity. Furthermore, the annotation of some subfamilies of DHH-DHHA1 proteins has been complicated by an overall lack of diagnostic sequence criteria to denote them. For example, many bacteria encode for proteins with DHH-DHHA1 domains, yet it is unclear how many should be annotated as NrnA proteins. For example, has a subset of NrnA proteins evolved to cleave only certain targeted sequences? Are some NrnA proteins differentially regulated? Moreover, are there subclasses of NrnA proteins that specialize in processing of signaling nucleotides? The full range of intracellular roles and physiologically relevant substrates remains to be explored for NrnA and NrnA-like proteins.

Prior investigations of NrnA-like proteins led to several different opinions on NrnA’s enzymatic activities and preferences. One possibility is that NrnA acts as a diribonuclease, akin to Orn. This speculation stems from the observation that overexpression of *B. subtilis nrnA* and *nrnB* in a *Pseudomonas aeruginosa* PA14 Δ*orn* strain resulted in restoration of the wild-type PA14 phenotype, as compared to PA14 Δ*orn*, which otherwise forms small colonies (13). Therefore, NrnA*_Bs_* and NrnB*_Bs_* either perform a similar function as Orn or exhibit functions that overlap with Orn. However, the published NrnA data are not uniform in their conclusions. For example, some studies have concluded that NrnA exhibits 3′-5′ exoribonucleolytic activity (15), while others have reported 5′-3′ exoribonucleolytic activity (19). Yet another study suggested that NrnA could exhibit both activities, preferentially cleaving longer RNA substrates from the 5′ end while targeting shorter RNA substrates from their 3′ terminus (21). If this were true, NrnA would represent a rare instance where an RNase exhibits dual polarity on oligoribonucleotide substrates. Yet other data argue that NrnA can act on ssDNA substrates (19). Furthermore, some stand-alone DHH-DHHA1 domain-containing proteins (e.g. *Staphylococcus aureus* Pde2, *Streptococcus pneumoniae* Pde2) were proposed to hydrolyze c-di-AMP to AMP in a two-step process via the linear intermediate AA, although there is not uniform agreement on that either (18,22). Whether NrnA-like proteins directly hydrolyze cyclic dinucleotide signaling molecules or possess an expanded range of RNA substrates has been further complicated by analyses of a *Mycobacterium tuberculosis* ‘NrnA-like protein’ that has been recently renamed CnpB (or Rv2837c) (23–26). While some data have argued that CnpB acts directly on short RNA substrates 2 - 5 nucleotides in length in addition to degrading a 24-mer RNA substrate (23), other studies have argued that CnpB specifically hydrolyzes c-di-AMP and c-di-GMP to nucleoside monophosphates in addition to linearizing 2′3′-cGAMP to GA (2′-5′) (24–26). Furthermore, cyclic nucleotide cleavage has also been reported for the *Mycobacterium smegmatis ‘*NrnA-like’ protein (27).

Yet other studies have linked ‘NrnA-like’ proteins to sulfur metabolism through their putative influence on pAp, which in some bacteria acts as an important carrier of sulfate. Specifically, NrnA*_Bs_* was shown to complement an *E. coli cysQ* mutant, which is auxotrophic for cysteine; in this strain, expression of *B. subtilis nrnA* restored cysteine prototrophy (15). Moreover, deletion of *B. subtilis nrnA* resulted in a slower growth rate in the absence of cysteine (15). Based on these observations, it was suggested that NrnA*_Bs_* may act as a CysQ-like phosphatase that dephosphorylates pAp to AMP. However, the reduction in *B. subtilis* growth rate for Δ*nrnA* was mild overall, hinting that there could still be a different enzyme that functions as a pAp phosphatase in *B. subtilis*.

Given the range of different claims that have been made on the activities and functions of NrnA-like proteins, it is difficult to categorically summarize exactly what enzymatic activity should be expected for a ‘NrnA’ protein. Simply put, there is great uncertainty about the substrate preferences and cellular functions of NrnA-like proteins. Importantly, the experimental conditions and assay formats have varied widely among these different studies. Given that NrnA*_Bs_* (and NrnB*_Bs_*) can complement *orn* deletion mutants (13–15), the simplest hypothesis is that all three proteins share exactly overlapping substrate requirements and overall cellular roles. But as summarized above, the available data on NrnA (and NrnB) paint a more confusing picture of what exactly these proteins are likely to be doing inside cells. Is NrnA a ‘diribonuclease’ akin to Orn? Or is it an RNase that specializes in processing either ‘short’ or ‘long’ RNA substrates? Or is NrnA a nuclease that processes both RNA and DNA substrates, as well as cyclic dinucleotides? And does NrnA process RNAs only in one direction, as do most RNase enzymes, or does it exhibit bidirectional abilities? To answer these questions, a variety of putative NrnA substrates must be assayed under a set of common reaction conditions. This could lead to a clearer definition of NrnA’s activity and could help discover distinct subclasses of NrnA-like proteins. This, in turn, could lead to identification of signature residues that guide substrate recognition for standalone DHH-DHHA1 family proteins.

In this study, we utilized a common set of reaction assays that closely resembled the assays that were recently used to re-examine Orn's diribonuclease activity (11). We reasoned that this approach would provide the best evidence for NrnA*_Bs_*’s substrate preferences, including the enzyme's directionality. To examine whether NrnA*_Bs_*’s activity could impact dinucleotide pools in vivo, we investigated whether the loss of *nrnA* or *nrnB* could affect cyclic di-GMP signaling. To directly investigate whether NrnA*_Bs_* and NrnB*_Bs_* could affect the abundance of other short RNAs *in vivo*, we examined the cleavage pattern of a radiolabeled 10-mer RNA in cellular extracts of wild-type, Δ*nrnA* and Δ*nrnB*. Finally, we also purified NrnA-like proteins from several species (*Streptococcus pyogenes* Pde2, *Enterococcus faecalis* NrnA, *Mycobacterium tuberculosis* CnpB, *Mycobacterium avium* CnpB, *Mycobacterium smegmatis* CnpB, and *Rhodococcus ruber* CnpB-like protein) and assayed them using the same reaction conditions as with NrnA*_Bs_*. This, we reasoned, would show whether different NrnA proteins behave similarly when compared under the same assay conditions. Together, our aggregate data show that NrnA proteins exhibit broader substrate preferences as compared to Orn. While Orn might preferentially process dinucleotides, NrnA acts as a housekeeping enzyme for degradation of short RNAs between 2 and 4 nucleotides in length and processes them from their 5′ terminus.

## MATERIALS AND METHODS

### Bacterial strains and culture conditions

All strains used in this study are listed in Supplementary Table S1. *E. coli* strains were grown in 2xYT supplemented with 100 μg/ml carbenicillin, and *B. subtilis* strains were grown in LB at 37°C, shaking with aeration (unless otherwise noted). When appropriate, *B. subtilis* strains were grown in the presence of 5 μg/ml chloramphenicol or 100 μg/ml spectinomycin. The methods for creating markerless deletions of *B. subtilis* Δ*nrnA, ΔnrnB*, and Δ*nrnA*Δ*nrnB*, and the integration of fluorescent c-di-GMP riboswitch-*yfp* reporter constructs have been previously described (13,28). To construct complementation strains, *nrnA_Bs_, nrnB_Bs_, orn_Vc_* and *cysQ_Ec_*were each PCR amplified from chromosomal DNA preparations. Sequences were subcloned via Gibson assembly (29) into the *amyE* integration vector pDR111, which harbors an IPTG-inducible promoter upstream of the target gene. Transformation of *B. subtilis* was performed using a previously described protocol (30). To construct *E. coli* strains for overexpression of targeted proteins, *nrnA_Bs_, nrnA_Ef_*, *pde2_Sp_*, *gdpP_(82__–__659)Bs_*, orn*_Ec_* and *cysQ_Ec_* were each PCR amplified from chromosomal DNA preparations. The sequences encoding *cnpB_Mt_*, *cnpB_Ma,_ cnpB_Ms,_ cnpB_Rr,_ disA_Bt_*, and *ppdk_cs_* were codon optimized for expression in *E. coli* and purchased from Integrated DNA Technologies. The different gene sequences were subcloned via Gibson assembly (29) into IPTG-inducible expression vector pAmr30, to yield an N-terminal 10xHis-SUMO tag that is cleavable by bdSENP1 protease (31). The *ppdk_cs_* and *gdpP_(82__–__659)Bs_*sequences were subcloned via Gibson assembly (29) into the IPTG-inducible expression vector pHis-parallel, to yield an N-terminal 6xHis tag. To create the point mutants of *nrnA_Bs_*, mutations were generated using the Q5 Site-Directed Mutagenesis Kit (NEB). *E. coli* XL10-Gold® (Agilent) was initially transformed with all plasmids, and sequences of all inserts were verified by Sanger sequencing. *E. coli* T7 Express (NEB) was transformed with all plasmids that were used for overexpression and purification of targeted proteins.

### Fluorescence microscopy and quantification

Single colonies were used to inoculate liquid minimal salts glycerol glutamate (MSgg) medium (32) and grown at 37°C with shaking overnight. The following morning, cultures of each strain were inoculated 1:50 in fresh MSgg medium and cultured, shaking at 37°C until reaching an OD_600_ of 1.0. Aliquots of each culture were placed on 1.5% low-melting-point agarose MSgg pads and allowed to dry for 10 min before inverting the pads and placing them on a glass bottom dish (Willco Wells). Cells were imaged at room temperature using a Zeiss Axio-Observer Z1 inverted fluorescence microscope equipped with a Rolera EM-C_2_ electron-multiplying charge-coupled (EMCC) camera, enclosed within a temperature-controlled environmental chamber. Quantification was performed with Oufti and FIJI software (33,34).

### Protein overproduction and purification

*E. coli* strains harboring expression vectors for 10xHis-SUMO-tagged protein sequences were cultured, shaking, overnight at 37°C. The following morning, the cultures were diluted in 500 ml fresh 2xYT supplemented with 100 μg/ml carbenicillin, 0.2% glucose (w/v) and 3 mM MgSO_4_. Cultures were grown shaking at 37°C until reaching an OD_600_ ∼0.4–0.8, at which point protein expression was induced with 1 mM IPTG. Cells expressing NrnA*_Bs_*, NrnA*_Ef_*, CnpB*_Mt_*, CnpB*_Ma_*, CnpB*_Ms_*, CnpB*_Rr_* and CysQ*_Ec_* were grown for an additional 2 h at 37°C. Cells expressing Pde2*_Sp_*, GdpP*_(82__–__659)Bs_*, DisA*_Bt_* or Orn*_Ec_* were removed from the 37°C incubator for expression. Cells expressing Pde2*_Sp_*, GdpP*_(82__–__659)Bs_*, DisA*_Bt_* were induced with 1 mM IPTG and cells expressing Orn*_Ec_*were induced with 0.4 mM IPTG. Pde2*_Sp_*, GdpP*_(82__–__659)Bs_*, DisA*_Bt_* or Orn*_Ec_* were grown at room temperature for 16 h. Cells were harvested by centrifugation and resuspended in 25 mM Tris–HCl pH 8.0, 300 mM NaCl, 0.25 mg/ml lysozyme, 0.01 mg/ml DNase I and 1 mM PMSF. Cells were lysed by sonication and insoluble material was removed by centrifugation. Clarified soluble lysates were incubated with cOmplete™ His-Tag Purification Resin (Roche) for 1 hour. After incubation, the resin was washed with 10 column volumes of Wash Buffer 1 (25 mM Tris–HCl, 300 mM NaCl, 10 mM imidazole, pH 8), followed by 5 column volumes of Wash Buffer 2 (25 mM Tris–HCl, 300 mM NaCl, 25 mM imidazole, pH 8). The following modification was made for GdpP*_(82__–__659)Bs_*, CnpB*_Ma_*, CnpB*_Ms_*, CnpB*_Rr_*, Orn*_Ec_* and NrnA(D80N D156N)*_Bs_*: all buffers also included 5% (v/v) glycerol. The following modification was made for DisA*_Bt_*: wash buffers were comprised of 62.5 mM Tris–HCl, and 750 mM NaCl. All proteins were eluted with 5 column volumes of 25 mM Tris–HCl, 300 mM NaCl, 250 mM imidazole, pH 8. Eluates were then dialyzed overnight against 25 mM Tris–HCl, 300 mM NaCl, pH 8.0. GdpP*_(82__–__659)Bs_*was dialyzed into 25 mM Tris–HCl, pH 8.0, 100 mM NaCl, 5% glycerol. GdpP*_(82__–__659)Bs_* was further purified using a Pierce™ Strong Anion Exchange Spin Column. The ion exchange column bound to GdpP*_(82__–__659)Bs_* was washed with 25 mM Tris–HCl, pH 8.0, 150 mM NaCl, 5% glycerol, and GdpP*_(82__–__659)Bs_* was eluted with 25 mM Tris–HCl, pH 8.0, 500 mM NaCl, 5% glycerol. The 6XHis tag was not removed from GdpP*_(82__–__659)Bs_*. NrnA*_Bs_*, NrnA*_Ef_*, CnpB*_Mt_*, CnpB*_Ma_*, CnpB*_Ms_*, CnpB*_Rr_*, DisA*_Bt_*, Orn*_Ec_* and CysQ*_Ec_* were subjected to 10xHis-SUMO tag cleavage by 10xHis-bdSENP1 in the presence of 2 mM DTT and 2 mM MgCl_2_ (30). Tag removal reactions were conducted on ice at 4°C for 4–12 h. Reactions were then incubated with cOmplete™ His-Tag Purification Resin (Roche) for 45 min to separate 10xHis-bdSENP1, free 10xHis-SUMO tag, and untagged proteins. Untagged proteins were recovered in the flow-through and dialyzed overnight against 25 mM Tris–HCl pH 8.0, 300 mM NaCl. Untagged proteins and GdpP*_(82__–__659)Bs_* were aliquoted, flash frozen in liquid nitrogen, and stored at –80°C.

### Preparation of whole cell lysates

Overnight cultures of *B. subtilis* strains were cultured shaking at 37°C. The following morning, cells were diluted into 500 ml fresh LB and grown at 37°C with shaking to OD_600_ ∼0.8. 250 μM IPTG was then added to the cultures and cells were grown for an additional 40 min. Cells were harvested by centrifugation and concentrated 10× in 25 mM Tris–HCl, 100 mM NaCl, pH 8.0. Following addition of 1 mM PMSF, cells were sonicated, and lysates were then aliquoted and stored at -80°C.

### Oligoribonucleotide labeling

Synthetic RNAs (2–7-mers) were purchased from TriLink Biotechnologies or Sigma-Aldrich. Each RNA was subjected to radioactive end-labeling or non-radioactive phosphorylation by T4 Polynucleotide Kinase (NEB). Each RNA was subjected to phosphorylation with equimolar concentrations of either [γ-^32^P]-ATP or ATP, T4 PNK and 1× T4 PNK Reaction Buffer. Reactions comprising a final concentration of either 0.5 μM 5′-[^32^P]-radiolabeled RNA or 2.0 μM phosphorylated RNA were incubated at 37°C for 40 min, followed by heat inactivation of T4 PNK at 65°C for 20 min. These labeling reactions were then utilized for subsequent RNase cleavage assays, as described in text. Due to the short length of RNA substrates, labeling efficiency was less than complete; residual [γ-^32^P]-ATP can be observed near the solvent front of denaturing PAGE gels.

### Synthesis of C-di-AMP

[^32^P]c-di-AMP was synthesized from reactions comprising 0.5 μM [α-^32^P]-ATP (Perkin Elmer), 0.5 μM unlabeled ATP, 50 mM Tris–HCl, 100 mM NaCl, 5 mM MgCl_2_, and 1 μM of purified DisA*_Bt_*. The reaction was incubated at 45°C for 5 h and then inactivated at 95°C for 10 min. The reaction was then centrifuged at 12 000 rpm in a 3 kDa MWCO NanoSep® centrifugal device for 20 min to remove DisA*_Bt_*. Conversion yield was determined by running an aliquot of the reaction on denaturing 20% PAGE and using a Cytiva Amersham Typhoon™ laser scanner platform. The intensity of radiolabeled c-di-AMP relative to the remaining ATP in the reaction was quantified using FIJI software (34).

### Oligoribonucleotide and C-di-AMP cleavage reactions

Phosphorylated RNA or c-di-AMP (1.0 μM), including trace amounts of the respective radiolabeled substrate, were subjected to cleavage *in vitro* at room temperature by 100 nM purified NrnA*_Bs_*, NrnA(D80N D156N)*_Bs_*, NrnA*_Ef_*, CnpB*_Mt_*, CnpB*_Mt_*, CnpB*_Mt_*, CnpB*_Mt_*, GdpP*_(82__–__659)Bs_* or Pde2*_Sp_*. These reactions were conducted in the presence of 25 mM Tris, pH 8.0, 300 mM NaCl and 200 μM MnCl_2_. At the appropriate times, aliquots of the reaction were removed and quenched in the presence of 150 mM EDTA on ice and heat inactivated at 95°C for 5 min. The reactions in Figure 2D were conducted by subjecting trace amounts of radiolabeled RNA molecules 2–7, and 10 nucleotides in length (GG, AGG, (A)_2_GG, (A)_3_GG, (A)_4_GG, (A)_5_GG, (A)_8_GG) to cleavage in vitro by 20 nM of purified NrnA*_Bs_*or Orn*_Ec_*in reactions containing either a physiological buffer comprised of 25 mM Tris, pH 8.0, 100 mM NaCl, 1 mM MgCl_2_, or 50 μM MnCl_2_, or a high manganese containing buffer composed of 25 mM Tris, pH 8.0, 100 mM NaCl, and 5 mM MnCl_2_. For reactions with *B. subtilis* whole cell lysates, trace amounts of radiolabeled RNA were added to lysates in the presence of 25 mM Tris–HCl pH 8.0, 300 mM NaCl, 200 μM MnCl_2_, and 25 mM MgCl_2_. At the appropriate times, aliquots of the reaction were removed and quenched in the presence of 150 mM EDTA on ice and heat inactivated at 95°C for 5 min. All cleavage reactions were separated on denaturing 20% PAGE containing 1× TBE and 4 M urea. The gels were imaged using Cytiva Amersham Typhoon™ laser scanner platform and analyzed for the appearance of truncated ^32^P-labeled products. The intensity of the radiolabeled nucleotides was quantified using ImageJ software (34).

### Differential radial capillary action of ligand assay (DRaCALA)

Apparent equilibrium binding reactions were performed by incubating trace amounts (∼1 nM) 5′-[^32^P]-dinucleotide with increasing concentrations of purified NrnA(D80N D156N)*_Bs_*in binding buffer (25 mM Tris–HCl pH 8.0, 300 mM NaCl, 200 μM MnCl_2_) for 30 min at room temperature. For competition assays, 1 μM purified NrnA(D80N D156N)*_Bs_* was incubated with trace (∼1 nM) 5′-[^32^P]AA in the presence of 10 or 100 μM unlabeled competitor, in binding buffer. Aliquots of all reactions were spotted onto a nitrocellulose membrane (GE) using a fixed replicator pin tool and allowed to air dry prior to being imaged on a Cytiva Amersham Typhoon™ laser scanner platform. Images were quantified using ImageJ software, and fraction bound was calculated based on previously described methods (34–36).

### Swimming motility assays

Plates of LB supplemented with 0.2% (w/v) agar with or without 100 μM IPTG were prepared and left to dry at room temperature overnight. Simultaneously, cells were grown overnight in LB with or without 100 μM IPTG. 4 μl of each stationary phase culture was stab-inoculated into the semi-solid agar plate and left to incubate at 30°C for 12 h. The diameter of the bacterial migration was measured with a ruler.

### 2-Aminopurine hydrolysis assays

To determine the polarity of NrnA*_Bs_* we used a fluorescence-based assay as described previously (37). This assay is based on the differential fluorescence output of the nucleotide analog 2-aminopurine. 2-Aminopurine shows reduced fluorescence when base-stacked with other nucleobases; however, free 2-aminopurine nucleotides exhibit increased fluorescence output. We monitored the fluorescence of 2-aminopurine generated from phosphodiester hydrolysis of synthetic 4-mer RNA substrates that were purchased from GE Healthcare Dharmacon. For this analysis, 100 nM of NrnA*_Bs_*was incubated with 10 μM of the RNAs containing a 2-aminopurine and a specific phosphorothioate modification. These reactions also contained 25 mM Tris–HCl, pH 8.0, 300 mM NaCl, 200 μM MnCl_2_. Reactions were conducted in a black 384-well plate using a Spectramax M5 plate reader, and fluorescence was measured every two min using an excitation wavelength of 310 and an emission wavelength of 375.

### pAp hydrolysis assays

NrnA-like proteins were assayed for pAp phosphatase activity using the Sigma-Aldrich^®^ Malachite Green Phosphate Assay Kit (MAK307). This assay was modeled on a previously described method, with slight modifications (38). A standard curve was generated using 1 mM free phosphate standard. All reactions were composed of 25 mM Tris–HCl pH 8.0, 300 mM NaCl, 100 μM pAp (Sigma), and either 200 μM of MnCl_2_ (for NrnA-like proteins), or 1 mM MgCl_2_ (for CysQ*_Ec_*) and conducted at room temperature. The indicated protein was added to its respective reaction and allowed to incubate for the indicated reaction time of either 5 or 30 min. Reactions were then quenched with one fourth of a volume of acidic malachite green dye solution and incubated for 30 min to allow for color development. Absorbance values at 620 nm (*A*_620_) were measured using an Agilent Cary 60 UV–Vis spectrophotometer and correlated against the standard curve to determine the concentration of inorganic phosphate released in each reaction. Several reactions were repeated under the same assay conditions but with higher concentration of NrnA, as indicated in Table 1.

**Table 1:** Concentration of free phosphate liberated by respective protein after incubation with 100 μM pAp detected by Malachite Green phosphate detection assay

	Concentration of phosphate liberated from pAp (mean ± SD) (μM)	
Protein	5 min	30 min
*B. subtilis* NrnA – 5 nM	ND	ND
*B. subtilis* NrnA – 50 nM	ND	9.7 ± 0.2
*B. subtilis* NrnA – 500 nM	9.3 ± 0.9	43.7 ± 1.7
*E. faecalis* NrnA – 5 nM	ND	ND
*S. pyogenes* Pde2 – 5 nM	ND	ND
*M. tuberculosis* CnpB – 5 nM	ND	ND
*E. coli* CysQ – 5 nM	6.3 ± 0.7	31.4 ± 1.2

ND, not detected

### Luciferase coupled enzyme assay: expression and purification of pyruvate phosphate dikinase

T7Express competent cells expressing His_6_-PPDK*_Cs_* were cultured to an OD_600_ of 0.6 at 37°C and induced with 1 mM IPTG for 2 h. Cells were harvested by centrifugation at 5000 rpm for 20 min. The cell pellets were weighed and resuspended in 1 g cell pellet per 10 mL of 25 mM Tris–HCl, pH 8.0, 1 M NaCl, 5% glycerol. To this mixture, PMSF was added to reach 1 mM and lysozyme to 0.25 mg/ml. Lysis proceeded by sonication on ice, then a 10% solution of polyethyleneimine was added to the sample to reach a final concentration of 0.5%. Lysates were clarified by two 15-minute rounds of centrifugation at 12 000 rpm. Next, PPDK*_Cs_* was precipitated with ammonium sulfate. Solid ammonium sulfate was gradually added to the clarified lysate to reach a final concentration of 40% saturation using the 0-degree Celsius convention. The lysate was then left to rock on ice for 30 min, after which it was centrifuged at 10 000 rpm for 10 min. After centrifugation, more solid ammonium sulfate was added to a final concentration of 50% saturation. The lysate was left to rock on ice for 30 min, and then centrifuged at 10 000 rpm for 10 min. At this point, the supernatant was decanted, and the precipitated PPDK*_Cs_* was resuspended in 25 mM Tris–HCl, pH 8.0, 300 mM NaCl, 5% glycerol. The protein sample was further purified by immobilization metal ion affinity chromatography using cOmplete™ His-Tag Purification Resin (Roche). The protein was incubated with equilibrated nickel resin for 1 h shaking gently on ice. Post incubation, the resin was washed with 10 column volumes of 25 mM Tris–HCl, pH 8.0, 300 mM NaCl, 5% glycerol 10 mM imidazole. Next, the resin was washed with 10 column volumes of 25 mM Tris–HCl, pH 8.0, 300 mM NaCl, 5% glycerol, 25 mM imidazole. Finally, PPDK*_Cs_* was eluted from the column using 5 column volumes of 25 mM Tris–HCl, pH 8.0, 300 mM NaCl, 5% glycerol, 250 mM imidazole. Purified PPDK*_Cs_* was dialyzed to remove excess imidazole. Then, purified PPDK*_Cs_* was concentrated using Corning® Spin-X® UF concentrators, and small aliquots of protein were flash frozen in liquid nitrogen and stored at –80°C.

### Luciferase coupled enzyme assay: determining the enzyme units of purified pyruvate phosphate dikinase

The enzymatic activity of NrnA*_Bs_*was assessed using a slightly modified continuous kinetic assay as described previously (39). For this analysis, AMP generated from phosphodiester hydrolysis of RNA substrates by NrnA*_Bs_*was converted to ATP by Pyruvate Phosphate Dikinase (PPDK*_Cs_*). In these reactions, ATP was then used by Luciferase enzyme to generate a luminescence signal. A 2× coupling buffer was used for these assays and was comprised of 100 mM Tris–HCl, pH 8.0, 200 mM NaCl, 2 mM phosphoenolpyruvic acid, 2 mM sodium pyrophosphate, 15 mM (NH_4_)_2_SO_4_. The 2× coupling buffer was filter sterilized and kept frozen. The enzymatic activity of dilutions of PPDK*_Cs_* were determined by monitoring the conversion of 100 μM AMP into ATP, using luminescence, and correlating ATP generation to a standard curve. Prior to the enzyme assay, 200 μl of fresh ATPlite substrate mix was added to 1 mL of the thawed 2X coupling buffer. The enzyme assays were conducted in the presence of the 1× coupling buffer containing the ATPlite mix, 10 mM MgCl_2_, 0.5 mM MnCl_2_, >50 units of PPDK*_Cs_* and the respective concentrations of the RNA substrates and purified NrnA*_Bs_* at 25°C. To ensure that there was a stable luminescence signal, we monitored the reactions containing all assay components except for the purified protein for 1–2 min prior to the addition of purified protein. For each concentration a blank was used that included all assay components except NrnA*_Bs_*, to control for background luminescence signal. Luminescence signals were measured continuously in a white 384-well plate using a Spectramax M5 plate reader. The luminescence signals were fit to standard curves to determine the concentrations of AMP released. For the degradation of AA, we divided the rates of AMP release by two since two molecules of AMP are liberated for each reaction.

### Mass spectrometry proteomics analysis

Wt and Δ*nrnA* strains were grown overnight and then subcultured followed by growth to an OD of 0.8. Cells were pelleted, supernatant removed and then resuspended in 5% SDS with 100 mM triethylammonium bicarbonate (TEAB). Protein abundance was acquired by using a Thermo Scientific Nanodrop 8000 spectrophotometer for sample normalization. Sample reduction in 200 mM DTT was performed for 1 hour at 57°C. Samples were then alkylated with 500 mM iodoacetamide for 45 min in the dark at RT and quenched by addition of 200 mM DTT. The S-Trap™ Micro Spin Columns (ProtiFi) preparation was performed according to manufacturer's protocols on 200 μg of protein (1:25, Trypsin/Lys-C:Protein Promega Cat.# V5073). Digested peptides were dried in a cold-trap vacuum centrifuge, and resuspended in 95:5 water:acetonitrile. 0.1% v/v formic acid was added to the samples and stored at –80°C until analysis. Samples were analyzed on a Thermo Orbitrap Eclipse Tribrid mass spectrometer equipped with a Thermo Ultimate 2500 UPLC and FAIMS unit operating under 3 different CVs (–50, –65 and –85). The liquid chromatography buffers used were mass spectrometry grade reagents (H_2_O and 0.1% formic acid for buffer A and 95% acetonitrile with 5% H_2_O and 0.1% formic acid for buffer B). A 75 cm Thermo Fisher Easy-Spray column was utilized for separation. A 200-minute gradient was used for separation at 0.3 μl/min flow rate (0 min 5% B, 10 min 10% B, 150 min 70% B, 158 min 80% B, 161 min 90% B, 171 90% B, 172 min 5% B, 200 min 5% B). MS1 was collected in the orbitrap at a resolution of 240 000 scanning from 250–2000 *m*/*z* with a standard AGC target and MIPS active. An intensity threshold of 2.04e^4^ was utilized along with charge state inclusion between 2 and 7. Dynamic exclusion was half to 60 s with a mass tolerance of 5 ppm. Data dependent MS2 were acquired in the ion trap, operating under the turbo scan rate with HCD activation at a 28% collision energy.

### Mass spectrometry data analysis

Data analysis was performed with Metamorpheous Version 0.0.320 (40). The database search was performed against the *B. subtilis* proteome from Uniprot (41) (Downloaded 5/11/2021). Search parameters included full Trypsin digest, 2 maximum miss cleavage events, 10 ppm precursor mass tolerance and 0.6 Da fragment tolerance. Dynamic modifications of oxidation on methionine and acetylation on the protein N-terminus were allowed. A static modification of carbamidomethylation was added to cystines. The FlashLFQ algorithm was utilized for relative label free quantification (42). *Q*-values and false discovery rate (FDR) were calculated using Percolator 3.0 (43). Results were filtered by requiring >0 unique peptides per protein, >1 PSMs per protein, and protein FDR ≤0.01.

### Collection of DHH-DHHA1 protein sequences and subsetting based on taxonomic class

Within the DHH phosphoesterase superfamily (Pfam: PF01368), there is a vast collection of protein subfamilies whose members can be distinguished by the presence of at least one additional (to the DHH domain), subfamily-specific domain. We were interested in instances where the DHH domain co-occurs with an adjacent DHH-associated domain, DHHA1 (Pfam: PF02272). A set of sequences whose domain architecture included the ordered combination of DHH and DHHA1 domains was collected by searching against the UniProtKB database using hmmsearch with a relaxed inclusion threshold, *E*-value ≤ 1E–1, and passing pre-trained profile hidden Markov models (HMMs) for each domain as the input. The resultant set consisted of only sequences in which there are subsequences that correspond to both DHH and DHHA1 domains. While the presence of these two domains were required, we permitted sequences that contained additional domains. Given that two subsequences are common across all sequences in the set, these subsequences can be concatenated for each sequence. Let the set of chimeric sequences be denoted by **S** = {*S_1_*, *S_2_*_, …,_*S_n_*}. In **S** (*n* = 63 390), we examined the organisms in which DHH/DHHA1 sequences are encoded and determined their prevalence within various taxonomic groups (classes). The DHH/DHHA1 proteins are phylogenetically pervasive. We selected two subsets of **S** from specific taxa—*Bacilli* (*n* = 11 068), and *Actinomycetia* (*n* = 1627)—to subsequently perform comparisons within subsets of sequences to assess sequence diversity that is driven by functional specialization rather than resulting from phylogeny (shared ancestry).

### CNM method-based approach to detecting sequence clusters

Suppose a subset of sequences *T* = {*n* ∈ **S***| n* is encoded in a specific taxon (class)}; additionally, the sequences in *T* are filtered using CD-Hit such that there is at most 80% pairwise sequence identity. We performed an all-against-all, pairwise BLAST search to quantify the similarity between pairs of unaligned sequences, denoted by *T_i_* and *T_j_*. The *E*-value of high scoring segment pairs from BLASTP were tabulated in a weighted adjacency matrix *I*; here, *I_ij_ i*s the *E*-value associated to the comparison of sequences *T_i_* and *T_j_* (for instance, *I_ij_* = 0 indicates *T_i_* and *T_j_* are identical). This measure of pairwise similarity is not symmetric, therefore we took the lower *E*-value between pairs such that *I* is symmetric. The matrix *I* was used to construct a weighted, undirected graph which layout is based on assigning weights to the edges that correspond to the distance between sequences (nodes), then implemented stress majorization to find an ideal configuration of nodes. Since values in *I* are the *E*-values, the edges between more similar sequences (indicated by lower *E*-values) are shorter. The neato utility from the Graphviz package was used for drawing graphs. Communities, or clusters, of similar sequences in the graph are detected by Clauset–Newman–Moore (CNM) modularity maximization. The greedy modularity communities function from the NetworkX Package was used for hierarchical and agglomerative partitioning of the sequences into distinct clusters, which we labeled based on the annotation of characterized representatives.

## RESULTS

### Deletion of *B. subtilis nrnA* results in accumulation of the dinucleotide GG and cyclic di-GMP

**Figure 1.**
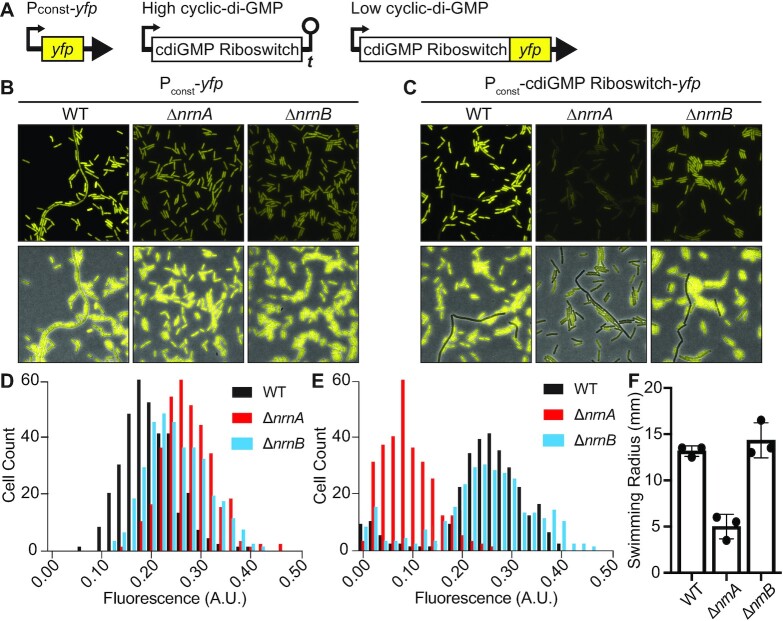
Intracellular detection of c-di-GMP abundance in *B. subtilis* 168 using a c-di-GMP-sensitive riboswitch reporter. (**A**) A schematic is shown for a Pconst-*yfp* reporter and a c-di-GMP-responsive riboswitch reporter, in the presence of high or low intracellular c-di-GMP levels. A similar riboswitch reporter was previously described (13). (**B**) Representative fluorescence microscopy images for cells containing the constitutively expressed YFP reporter Pconst-*yfp*. These strains consisted of wild-type (WT) *B. subtilis*, *ΔnrnA*, or *ΔnrnB*. (**C**) Representative microscopy images of the c-di-GMP-responsive riboswitch reporter expressed in WT, *ΔnrnA* or *ΔnrnB* strains of *B. subtilis*. The top row of pictures shows only fluorescence data while the bottom row shows the fluorescence data overlayed over phase contrast images. (D, E) Histograms of the quantification of average fluorescence intensity of individual cells for *B. subtilis* 168 WT, *ΔnrnA* or *ΔnrnB*, containing eitherPconst-*yfp* (**D**) or the c-di-GMP responsive riboswitch reporter (**E**) (*n* = ∼300). (**F**) Swimming motility analysis of WT, Δ*nrnA* or *ΔnrnB* strains of *B. subtilis* 168. Growth medium contained 0.2% agar and was analyzed after a 12-h incubation period at 30°C.

The recent re-evaluation of Orn's function as primarily acting as a diribonuclease (11) led us to reconsider the individual roles NrnA*_Bs_* and NrnB*_Bs_* have *in vivo*. Orn's role as a diribonuclease has implications in cyclic di-guanosine monophosphate (c-di-GMP) signaling, as the dinucleotide GG is an intermediate in the two-step conversion of c-di-GMP to two GMP molecules. The ability of *B. subtilis nrnA* and *nrnB* to complement the *E. coli* and *P. aeruginosa* Δ*orn* mutants suggested that these enzymes are responsible for GG cleavage in *B. subtilis* in a manner that is analogous to Orn function (13–15). It has been shown previously that an increase in GG leads to a concomitant increase in c-di-GMP through feedback inhibition. Previously, a double Δ*nrnA*Δ*nrnB* mutant was assayed for c-di-GMP levels using a fluorescent c-di-GMP riboswitch reporter (13,28,44). This revealed that levels of c-di-GMP, and consequently GG, were higher in this strain compared to wild-type. However, in this study, we sought to determine the relative contributions of GG turnover from NrnA*_Bs_* and NrnB*_Bs_*individually. Therefore, c-di-GMP levels were assayed using the same c-di-GMP-responsive riboswitch-*yfp* reporter but for *B. subtilis* wild-type, *ΔnrnA*, or Δ*nrnB* cellular backgrounds (Figure 1A). The c-di-GMP-responsive riboswitch decreases downstream gene expression in response to an increase in c-di-GMP; therefore, YFP expression is inversely coupled to c-di-GMP abundance in this strain. Assessment of a constitutive *yfp* control reporter showed mild to no differences in fluorescence between the wild-type strain and the single Δ*nrnA* or Δ*nrnB* mutants (Figure 1B, D). In agreement with our prior observations (28), assessment of the c-di-GMP reporter in a wild-type *B. subtilis* population showed a bimodal distribution of fluorescence where one population exhibited high c-di-GMP (low YFP fluorescence) and the second exhibited low c-di-GMP (high YFP fluorescence) (Figure 1C, E). In the Δ*nrnA* background, the riboswitch reporter exhibited very low fluorescence compared to the wild-type, indicating that cyclic di-GMP levels, and, by extension, GG levels, are higher in this strain. This observation is consistent with a role in turnover of GG for NrnA*_Bs_*. However, a bimodal distribution of fluorescence was observed from the riboswitch-*yfp* reporter in the Δ*nrnB* strain, similar to wild-type (Figure 1C, E). This suggests that if NrnB_Bs_ does cleave GG during late exponential phase of growth (OD_600_ ∼1.0), accumulation of the dinucleotide in the absence of NrnB*_Bs_* is not enough to promote feedback inhibition of enzymes that linearize c-di-GMP. Alternatively, NrnB*_Bs_*might not be expressed during exponential growth phase.

To further investigate how NrnA can affect *B. subtilis* c-di-GMP pools, we assessed motility, which is regulated by c-di-GMP in many bacteria (45). Prior experiments in *B. subtilis* have shown that elevated intracellular c-di-GMP levels lead to an inhibition of swarming motility—a social form of surface migration (46,47). *B. subtilis* uses flagella for swimming through liquid as well (47). Fortifying media with 0.2% agar allows the pores in the agar to be sufficiently large enough to discourage swarming over the surface, but rather, permit swimming. The Δ*nrnA* mutant exhibited a clear swimming defect compared to wild-type, further suggesting that intracellular c-di-GMP levels are elevated in this strain (Figure 1F). In contrast, the Δ*nrnB* mutant had a phenotype similar to wild-type. Together, these data show that NrnA*_Bs_* but not NrnB*_Bs_* is important for maintaining cellular GG pools during exponential phase growth.

### NrnA*_Bs_* cleaves RNA substrates 2-4 nucleotides in length *in vitro*

**Figure 2.**
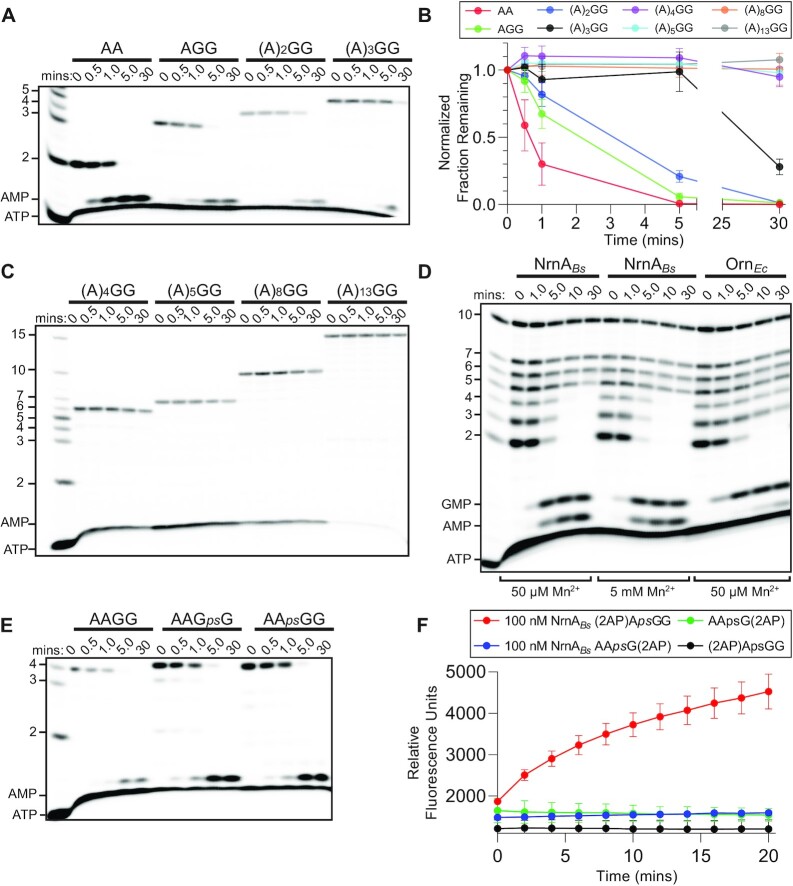
*B. subtilis* NrnA has a preference for hydrolyzing short RNAs 2–4 nucleotides in length, and acts with a 5′ - 3′ polarity. (A-C) 1 μM of RNA substrates containing trace amounts of ^32^P-radiolabeled RNA were incubated with 100 nM of purified NrnA_Bs_. The RNA substrates ranged from 2-7, 10 or 15 nucleotides in length.Aliquots were removed at various time points and resolved by denaturing PAGE. The cleavage data were graphed as the normalized intensity of the initial substrate, plotted as the average and SD of three independent experiments in (A and C). (D) Trace amounts of different ^32^P-radiolabeled RNA substrates that ranged in size (including 2–7, and 10-mer RNAs) were simultaneously incubated with 20 nM of purified NrnA*_Bs_* or Orn*_Ec_*_._These reactions also contained either low or high concentrations of manganese. Aliquots were removed at time intervals and quenched in 150 mM EDTA and 4 M urea. Degradation products were resolved by denaturing PAGE. (E) In these reactions, some RNA substrates contained a non-hydrolyzable, phosphorothioate-modified backbone (placement of the linkage represented by *ps*). 1 μM of RNA was incubated with trace amounts of ^32^P-radiolabeled RNA and 100 nM of purified NrnA_*Bs*_. Aliquots were removed at various time points and resolved by denaturing PAGE. (F) Fluorescence emission changes of 10 μM RNA substrates containing both a 2-aminopurine and an internal phosphorothioate linkage, upon incubation in the presence or absence of 100 nM NrnA.

To fully understand its length preferences, purified NrnA*_Bs_* was tested for cleavage of different RNA substrates. NrnA*_Bs_*was incubated with 5′-^32^P-radiolabeled oligoribonucleotides of varying lengths and at substrate concentrations that exceeded enzyme concentration (10:1). These reactions also contained divalent cations for supporting catalysis. The products of these reactions were analyzed by urea-denaturing 20% polyacrylamide gel electrophoresis (PAGE). Analysis of NrnA*_Bs_*showed that it fully processed the dinucleotide AA into nucleoside monophosphates in 5 min (Figure 2A, B). Importantly, NrnA*_Bs_*also showed similar activity against a 3-mer RNA (AGG) within the same time frame. While some processing of a 4-mer (AAGG) was observed in the first 5 min, the rate of cleavage appeared to be slower than that of the 3-mer or dinucleotide. This contrasts with a 5-mer RNA, which remained generally unprocessed after the first 5 min but was processed over longer time points.

### NrnA*_Bs_* does not cleave longer RNA substrates *in vitro*

Using the same reaction conditions that were used to test short RNA substrates, we incubated NrnA*_Bs_*with 6-mer, 7-mer, 10-mer and 15-mer substrates. This revealed no detectable cleavage activity against the longer substrates (Figure 2B, C). Indeed, when NrnA*_Bs_* was simultaneously incubated with radiolabeled RNAs of 2–7, and 10 nucleotides in length, only the RNAs from 2–4 nucleotides in length were processed (Figure 2D). These data mildly conflict with prior reports that NrnA*_Bs_* degrades longer nucleic acid substrates greater than four nucleotides in length (15,19,21). Our data strongly suggest that NrnA*_Bs_*preferentially degrades short RNAs four nucleotides and smaller in length, and that the previously reported activities against longer substrates may have arisen due to the stochiometric excess of enzyme and/or long incubation times used in the prior assays. Therefore, we speculate that long RNA processing is not likely to be a biologically relevant function of NrnA*_Bs_*. Additionally, these data indicate that the processing of 2–4 mer RNAs by NrnA*_Bs_* is only modestly affected by increasing the concentration of Mn^2+^. Interestingly, when Orn*_Ec_*was simultaneously incubated with radiolabeled RNAs 2–7, and 10 nucleotides in length, dinucleotides are rapidly processed, and only minimal trinucleotide processing was seen over the longer time points (Figure 2D). In total, these data demonstrate that under reaction conditions where Orn*_Ec_* displays diribonuclease activity, NrnA*_Bs_* instead displays a specific preference for short RNAs between 2–4 nucleotides in length.

### NrnA*_Bs_* cleaves RNA substrates from their 5′ terminus

Interestingly, processing of short RNAs by NrnA*_Bs_* resulted in release of nucleoside monophosphates but did not result in the appearance of degradation intermediates. This suggests that cleavage activity does not occur in a 3′-5′ direction as intermediates would be expected for RNAs containing a radiolabeled phosphate at the 5′ terminus. This observation agrees with certain prior investigations on NrnA*_Bs_* that showed that cleavage of 5′-^32^P-AAA rapidly resulted in release of ^32^P-AMP (21), which would be the expected outcome if NrnA*_Bs_* were removing the terminal phosphate from the 5′ direction. Yet, NrnA proteins have also been described as a 3′ to 5′ exonuclease (15,21,23,48). Therefore, NrnA*_Bs_’*s directionality remains unclear. To test the directionality of NrnA*_Bs_*cleavage under our standard assay conditions, we assessed NrnA*_Bs_* activity against 5′-^32^P-radiolabeled 4-mer RNAs that contained a putatively nonhydrolyzable phosphorothioate linkage at different locations. As anticipated, cleavage of a native 4-mer resulted in release of ^32^P-AMP (Figure 2E). However, NrnA*_Bs_*also released ^32^P-AMP upon incubation with a 4-mer containing a phosphorothioate linkage at its 3′ terminus. Furthermore, cleavage of a 4-mer with a phosphorothioate linkage in the middle bond of the RNA (^32^P-AA*ps*GG) did not result in ^32^P-AA*ps*G, which would have been expected if NrnA*_Bs_*were cleaving from the 3′ direction, but instead again resulted in release of ^32^P-AMP (Figure 2E). The 5′-A*ps*AGG substrate could not be assessed in this analysis, as it was not successfully radiolabeled by polynucleotide kinase. However, since this assay did not rule out the possibility that NrnA could process through the phosphorothioate linkage, we utilized a second approach to assess termini selectivity. Specifically, we determined whether NrnA would release a 2-aminopurine (2AP) nucleotide from either the 5′ terminus or 3′ terminus for 4-mer RNA substrates containing an internal phosphorothioate linkage (*i.e*. (2AP)A*ps*GG or AA*ps*G(2AP)). Free 2AP is known to exhibit an increased level of intrinsic fluorescence relative to 2AP contained within an RNA polymer, due to base stacking of the latter. When NrnA*_Bs_* was incubated with (2AP)A*ps*GG, an increase in 2AP fluorescence was observed over time (Figure 2F). In contrast, no fluorescence increase was observed upon incubation of NrnA*_Bs_* with AA*ps*G(2AP). Taken together, these assays suggest that NrnA*_Bs_* is exclusively a 5′-3′ exoribonuclease for short RNA substrates.

### NrnA*_Bs_* preferentially binds short RNAs

**Figure 3.**
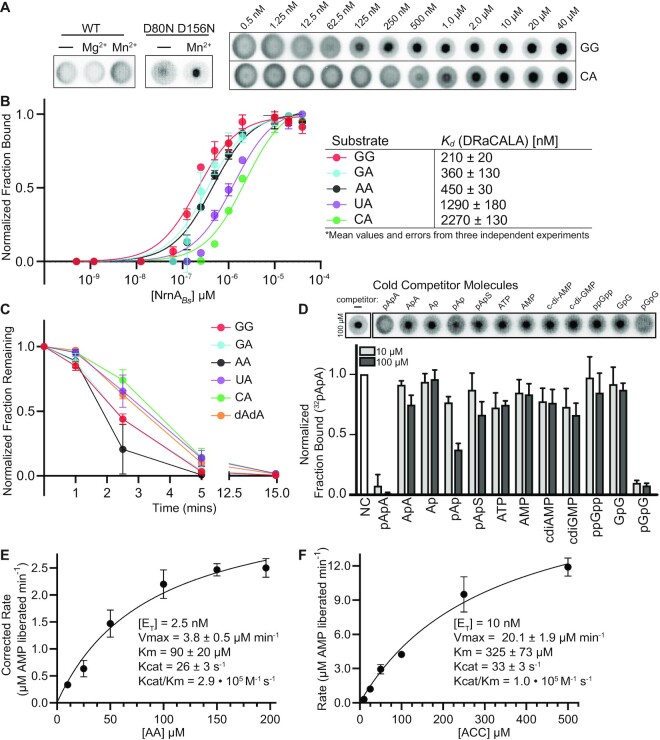
*B. subtilis* NrnA possesses a dinucleotide substrate length preference with a slight preference for 5′ purine residues. (**A**, **B**) DRaCALA, as described elsewhere (35), was used to measure binding of different radiolabeled dinucleotides to the catalytically inactive mutant NrnA*_Bs_* D80N D156N. (**C**) For cleavage assays, 1 μM of the indicated dinucleotide substrate was incubated with trace amounts of ^32^P-radiolabeled RNA and 100 nM of NrnA Products were removed at time intervals and resolved by denaturing PAGE. Quantification of the normalized radioactive intensity of the initial substrate was plotted as the average and SD of three independent experiments. (**D**) Substrate binding competitions were conducted by DRaCALA. 1 μM of purified NrnA_*Bs*_ D80N D156N was bound to ∼1 nM of ^32^P labeled AA, and subsequently incubated with either 10 or 100 μM of the indicated unlabeled competitor molecules. The fraction bound was normalized to the maximum binding exhibited by NrnA_*Bs*_ D80N D156N to ^32^P-radiolabeled AA. (D, E) Kinetic analysis of *B. subtilis* NrnA was performed using a previously published continuous kinetic assay (39), with slight modifications. The continuous coupled enzyme assay depended on the liberation of AMP from the RNA substrate by NrnA_Bs_, followed by the conversion of AMP to ATP by PPDK_Cs_, which was then used by luciferase to generate a pulse of light. Since the hydrolysis of AA results in the generation of 2x AMP molecules, the initial rates for AA hydrolysis were divided by 2. Substrate degradation was restricted to 10% or less, and a blank containing all reaction materials except for NrnA*_Bs_* was included as a control for background luminescence. (B–F) All data was plotted as the average and SD of three independent experiments.

We next sought to correlate NrnA*_Bs_*cleavage preferences with binding affinities by using Differential Radial Capillary Action of Ligand Assay (DRaCALA) (35). Structural analyses of NrnA*_Bs_*and other NrnA-like proteins have demonstrated that the NrnA active site incorporates four aspartate residues (NrnA*_Bs_*D24, D26, D80, D156), and three histidine residues (NrnA*_Bs_*H20, H103, H104). Catalysis by NrnA and other DHH domain-containing proteins are coordinated by a two-ion metal mechanism, in which manganese is the preferred ion for catalysis (19,21,48), although magnesium can also support catalysis *in vitro*. Herein, we incubated NrnA*_Bs_*with 5′-radiolabeled AA in the presence of magnesium or manganese and assayed for protein-RNA complexes by DRaCALA (Figure 3A, B). We did not observe evidence of binding activity, most likely because the RNase enzyme is active under these conditions, thereby processing the dinucleotides into nucleoside monophosphates. We also did not observe binding activity of 5′-radiolabeled AA with NrnA*_Bs_* that had been treated with the divalent cation chelator EDTA (Figure 3A). NrnA*_Bs_*is not catalytically active in the presence of excessive EDTA; therefore, the lack of RNA-binding activity under these conditions may suggest that divalent ions are required for substrate binding. We therefore sought to investigate binding of AA to a catalytically inactive mutant. To that end, we mutated two of the highly conserved aspartate residues to asparagine (D80N D156N) (49). This mutant protein exhibited no detectable RNase cleavage activity (Supplementary Figure S1B), although it demonstrated binding activity to AA when incubated with supplemental divalent metals (Figure 3A). A survey of varying dinucleotide concentrations revealed that NrnA*_Bs_*D80N D156N bound GG best, with an equilibrium dissociation constant (*K*_d_) of 210 nM, while other dinucleotides (GA, AA, UA and CA) exhibited modestly poorer binding affinities (Figure 3A, B). The poorest binding affinities were observed for UA and CA, which were roughly an order of magnitude higher than GG. We were surprised at the differences in these apparent equilibrium binding affinities; therefore, we tested for their cleavage by wild-type NrnA*_Bs_* in vitro (Figure 3C). This showed that the dinucleotides were all equally good substrates for NrnA*_Bs_*, with only minor differences in their rates of cleavage. As it was previously reported that NrnA*_Bs_* could exhibit activity against single-stranded DNAs (19), we also compared cleavage of a DNA dinucleotide alongside the RNA dinucleotides. This revealed that NrnA*_Bs_* is indeed fully active against a DNA dinucleotide substrate (Figure 3C). From these aggregate data, we conclude that NrnA*_Bs_* generally cleaves dinucleotides but with a modest preference for purine-containing substrates.

We then exploited the binding activity of the double aspartate mutant to survey for recognition of different putative substrates. Specifically, we determined the fraction bound of radiolabeled AA when co-incubated in the presence of unlabeled (10 or 100 μM) putative substrates (Figure 3D). As anticipated, the unlabeled dinucleotides AA and GG competed well against 5′-radiolabeled AA. However, ApA and GpG did not compete for binding, suggesting that a 5′ phosphate is likely to be critical for binding. Indeed, an adenosine with phosphates at its 5′ and 3′ termini (pAp) was unable to compete at 10 μM but could partially compete against AA when supplied at 100 μM, suggesting that pAp exhibits reduced but measurable binding affinity for NrnA*_Bs_*. pAp is a cytoplasmic carrier of sulfate when converted to pApS (50); however, pApS was unable to compete for binding to NrnA*_Bs_*. Other phosphorylated mononucleotides (e.g. ATP, AMP) were also unable to compete for binding. Finally, cyclic di-nucleoside monophosphates (c-di-AMP and c-di-GMP) were unable to compete against binding of AA. These data reveal that the NrnA*_Bs_* binding pocket is best suited for canonical dinucleotides and is unlikely to act directly on nucleotide signals.

### Kinetic analysis of dinucleotide and trinucleotide substrates against NrnA*_Bs_*

While NanoRNase enzymes have been argued to cleave a range of short RNA substrates, the Orn enzyme is suspected in some bacteria to strongly prefer cleavage of dinucleotides under physiological conditions. When we correspondingly tested the cleavage of NrnA*_Bs_* against 2–5 mer RNA substrates at potentially physiologically relevant concentrations the rate of cleavage for 2–4 mers were similar (Supplementary Figure S2). The concentrations used in this analysis likely represent a second order rate equation which could be useful for determining the substrate specificity of NrnA*_Bs_*. From these data, we speculated that NrnA*_Bs_* might possess a slight dinucleotide specificity. Previous reports regarding the specificity of NrnA-like proteins have led to different conclusions as to whether NrnA-like proteins are specific for dinucleotides or other short RNA substrates. NrnA*_Bs_* was originally reported to possess a preference for 3-mer substrates in cleavage assays using 5′-Cy5 modified oligonucleotides (15). However, our data in Figure 2D strongly suggests that the 5′ phosphate plays a crucial role in ligand binding. Therefore, these data led us to believe that the 5′-Cy5 modified RNAs could have influenced the reported enzyme activity. Also, the NrnA-like protein from *Thermatoga maritima* was previously reported to possess a dinucleotide specificity based on the protein's reduced activity in cleavage assays against the trinucleotide AAA (49). Hence, we wanted to acquire additional quantitative data directly comparing the kinetics of cleavage for a dinucleotide and trinucleotide substrate. To that end, we modified a luminescence-based, coupled enzyme assay for measuring release of AMP from either an AA or ACC substrate (see Materials and Methods). These data showed that NrnA*_Bs_* displayed a Michaelis–Menten kinetic profile (Figure 3E, F). This revealed that the Km values differed modestly between the two RNAs (0.090 versus 0.325 mM). However, a direct comparison of the specificity constants (*k*_cat_/*K*_m_) for these reactions did not differ significantly. Therefore, NrnA*_Bs_* acts efficiently against both di- and trinucleotide RNA substrates.

### Substrate preferences of NrnA*_Ef_*and Pde2*_Sp_* closely resemble NrnA*_Bs_*, but not CnpB*_Mt_*

**Figure 4.**
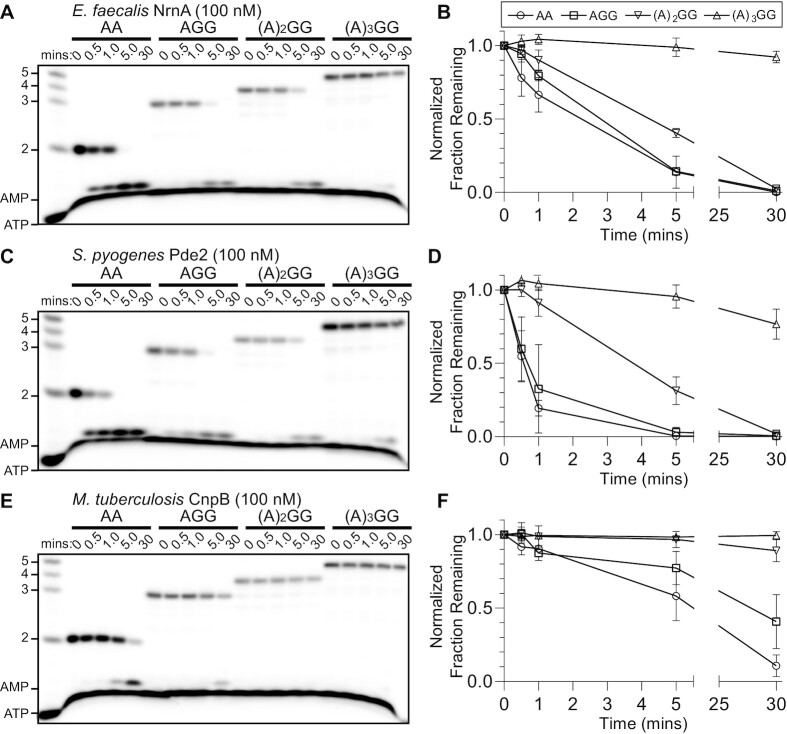
Degradation profiles of short RNAs incubated with NrnA-like proteins. (**A, C, E**) 100 nM of the respective protein was incubated with 1 μM of RNA substrate, 2–5 nucleotides in length, containing trace amounts of ^32^P-radiolabeled substrates. Aliquots from reactions were removed at the indicated times and quenched in 150 mM EDTA and 4 M urea, and degradation products were resolved by denaturing PAGE. (**B, D, F**) The fraction of initial substrate was plotted over time as the average and SD of three independent experiments.

Several different catalytic functions have been attributed to the NrnA-like *Mycobacterium tuberculosis* CnpB protein, including but not limited to cleavage of short and long RNA substrates and processing of c-di-AMP (23,25,26,51). The streptococcal Pde2 protein has been implicated in processing of c-di-AMP but has not yet been assessed for cleavage of linear RNAs (52). Yet, the sequence identity between these proteins and NrnA*_Bs_* are reasonably high at 45% for *S. pyogenes* Pde2 and 25% for the *M. tuberculosis* CnpB. Therefore, we wanted to directly compare the substrate preferences of these enzymes (CnpB*_Mt_*, Pde2*_Sp_*) against NrnA*_Bs_* using the same substrates and reaction conditions. To further extend these analyses, we also included an NrnA-like proteins from *Enterococcus faecalis*, which has not yet been investigated. Herein, we incubated purified NrnA*_Bs_*, CnpB*_Mt_*, NrnA*_Ef_* and Pde2*_Sp_* with a variety of short RNA substrates. Each protein was incubated with 5′-radiolabeled oligoribonucleotides between 2–5 nucleotides in length and the reaction products were resolved by 20% denaturing PAGE (Figure 4). From these data, it is apparent that NrnA*_Ef_*, and Pde2*_Sp_* process short RNAs between 2–4 nucleotides in length, remarkably like NrnA*_Bs_*. Moreover, both proteins fully processed the dinucleotide AA into nucleoside monophosphates within the same approximate time frame as NrnA*_Bs_* (Figure 4A–D). A trinucleotide RNA substrate was also fully processed by both proteins within a time frame that resembles dinucleotide substrate. Interestingly, while CnpB*_Mt_* was able to fully process AA to NMPs within 30 min, the overall rate of cleavage appeared to be slower than that of the other proteins (Figure 4E, F). Also, an assessment of longer substrates showed essentially no cleavage activity for CnpB*_Mt_* against them (Supplementary Figure S3). Taken together, the NrnA*_Ef_*, and Pde2*_Sp_* proteins appear to exhibit substrate preferences that closely resemble NrnA*_Bs_*, while CnpB*_Mt_* exhibits lowered activity against linear RNAs.

### NrnA*_Bs_* does not act on cyclic di-AMP

While our binding analysis of the NrnA*_Bs_*D80N D156N mutant suggested that the protein was not likely to bind c-di-NMPs, other DHH-DHHA1 domain-containing proteins have been observed to specifically hydrolyze c-di-AMP; therefore, we wanted to directly test for this activity using the same reaction conditions as for cleavage of small linear RNA substrates. GdpP is one of the DHH-DHHA1 domain-containing proteins that has been implicated in cleavage of c-di-AMP. Unlike NrnA, which only contains the DHH-DHHA1 domains, GdpP also includes an N-terminal transmembrane domain, a heme-binding PAS domain, and a degenerate GGDEF domain (53). However, in contrast to the architectural complexity of GdpP, *M. tuberculosis* CnpB only contains the DHH-DHHA1 domains but has also been reported to exhibit c-di-NMP phosphodiesterase activity (25,26). Yet, this observation has been debated and, akin to NrnA*_Bs_*, multiple other activities have been attributed to CnpB*_Mt_*, such as cleavage of RNAs from 2–5 nucleotides in length, with an Orn-like preference for dinucleotides (23). Furthermore, despite showing c-di-AMP hydrolysis activity *in vitro*, it was recently reported that expression of *cnpB* in vivo is entirely independent of c-di-AMP (54). In addition to CnpB, Pde2 has also been linked to homeostasis of cyclic di-NMPs. Several studies have suggested that Pde2 can process c-di-AMP in vitro and can preferentially hydrolyze AA to AMP (18,52).

**Figure 5.**
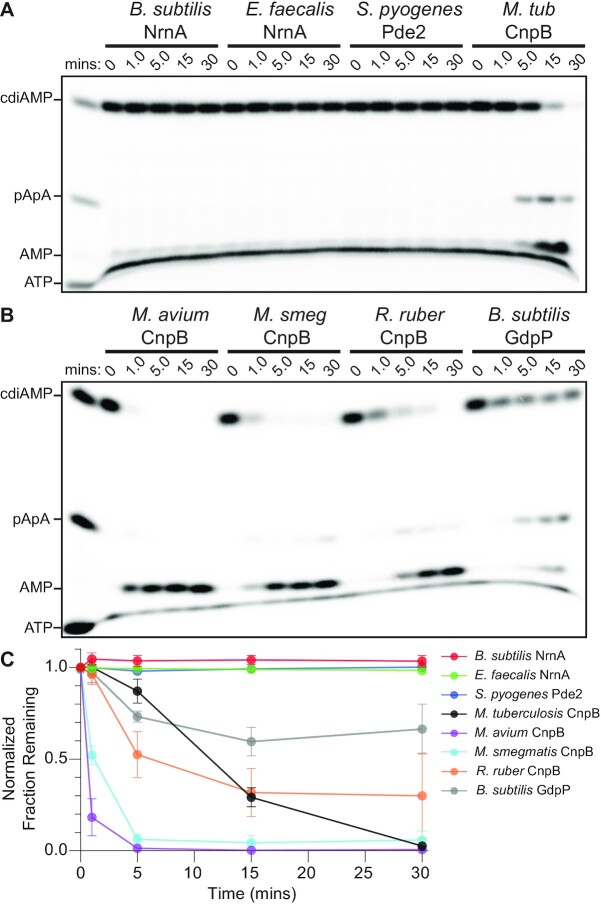
NrnA-like proteins differ in their capacities to process c-di-AMP. (**A**, **B**) 100 nM of the respective protein was assayed for c-di-AMP phosphodiesterase activity against 1 μM of substrate containing trace amounts of ^32^P-labeled c-di-AMP. Aliquots were removed from the individual reactions and were quenched in 150 mM EDTA and 4 M urea. Degradation products from the reactions were resolved by denaturing 20% PAGE. (**C**) The fraction of initial substrate was plotted over time as the average and SD of three independent experiments.

To determine whether NrnA-like proteins had the ability to cleave c-di-AMP, NrnA*_Bs_*, CnpB*_Mt_*, NrnA*_Ef_* and Pde2*_Sp_* were purified and then tested for cleavage of ^32^P-radiolabeled c-di-AMP. All four proteins were assayed under the same conditions, which resembled the reaction conditions employed for analysis of dinucleotides. When the products of these reactions were analyzed by urea-denaturing 20% polyacrylamide gel electrophoresis (PAGE), only CnpB demonstrated an ability to directly hydrolyze c-di-AMP to AMP (Figure 5A, C); no cleavage activity was detected for NrnA*_Bs_*, NrnA*_Ef_* and Pde2*_Sp_*. To ascertain whether this activity may be representative of CnpB-like proteins, we also purified CnpB homologs from *Mycobacterium smegmatis, Mycobacterium avium* and *Rhodococcus ruber* and then assayed for cleavage of c-di-AMP. As a positive control, we purified and analyzed the cytoplasmic portion of *B. subtilis* GdpP, which is known to hydrolyze c-di-AMP (53). All CnpB-like proteins exhibited robust hydrolysis of c-di-AMP (Figure 5B). Together, these data suggest that CnpB*_Mt_* may broadly feature active site residues that make it functionally different from other NrnA-like proteins, which do not directly process c-di-AMP.

### An assessment of 3′phosphoadenosine-5′-phosphate (pAp) cleavage activity

It has also been reported that NrnA*_Bs_* and CnpB*_Mt_* might act as pAp phosphatases, which affect sulfur metabolism by altering homeostasis of 3′-phosphoadenosine-5′-phosphosulfate (pApS) (15,23). However, we found that pAp and pApS were only moderate and poor binding competitors for the NrnA*_Bs_* enzyme, respectively (Figure 3D). Therefore, to directly investigate this question, we surveyed each of the NrnA-like proteins for pAp hydrolysis under the same reaction conditions and alongside a positive control. Specifically, we purified *E. coli* CysQ, a known pAp phosphatase, as a positive control for these assays (10). Then, purified NrnA*_Bs_*, CnpB*_Mt_*, NrnA*_Ef_* and Pde2*_Sp_* were each assayed for pAp phosphatase activity in the presence of divalent cations. Each protein was incubated with 100 μM pAp and the concentration of free phosphate generated in the reaction was determined via a malachite green detection assay. Under these conditions, 5 nM CysQ*_Ec_* generated 6.3 and 31.4 μM free phosphate in 5 and 30 min, respectively (Table 1). However, free phosphate was not detectable following incubation with pAp for any of the NrnA-like proteins at these enzyme concentrations (NrnA*_Bs_*, CnpB*_Mt_*, NrnA*_Ef_* and Pde2*_Sp_*). However, when the concentration of NrnA*_Bs_* was increased 10× and 100X, phosphate release increased. This observation may explain previously published reports on pAp hydrolysis (15,23). Based on the general lack of robust phosphatase activity coupled with the inability of pAp to compete for binding against linear AA (as seen in Figure 3D), we speculate that NrnA-like proteins are unlikely to preferentially act on pAp under physiological conditions.

### NrnA*_Bs_* is required for processing of short RNAs *ex vivo*

**Figure 6.**
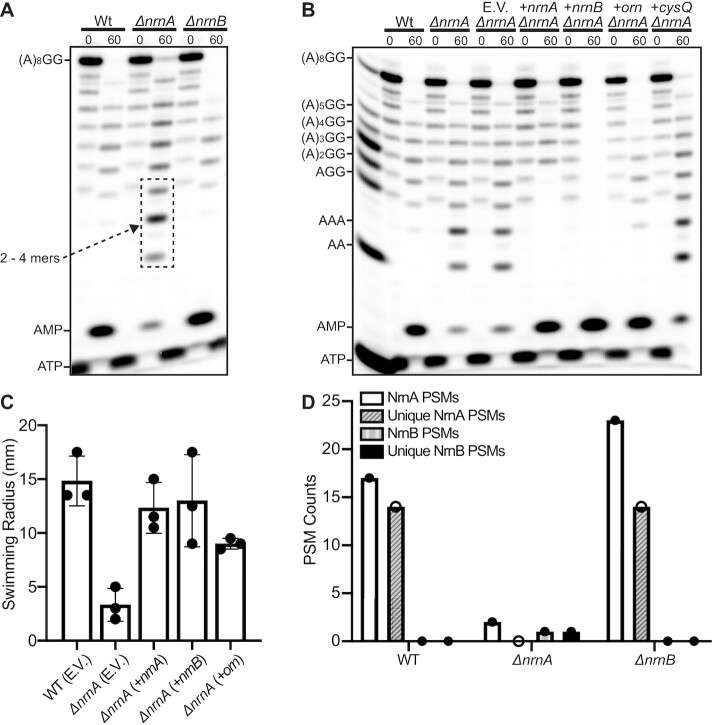
Cleavage of short RNAs by *B. subtilis* cellular lysates. (**A**) Whole cell lysates of WT*, ΔnrnA*, Δ*nrnB* or (**B**) *ΔnrnA* complementation strains containing IPTG-inducible expression of *nrnA_Bs_, nrnB_Bs_, orn_Vc_*, or *cysQ_Ec_* were harvested during vegetative growth and incubated with 1 μM of a 5′-radiolabeled 10-mer RNA. Aliquots were removed at various time intervals. (A and B) Degradation products from cellular lysates were resolved by denaturing PAGE. (**C**) Swimming motility analysis of WT or *ΔnrnA* complementation strains that contained IPTG-inducible expression of *nrnA_Bs_*, *nrnB_Bs_*, or *orn_Vc_*. Motility plates contained 0.2% agar and were analyzed after a 12-hour incubation period at 30°C. E.V. indicates that the strain was transformed with an empty vector control. (**D**) Untargeted mass spectrometry analysis of wild-type, Δ*nrnA* or Δ*nrnB* strains yields high confidence peptide spectral matches attributed to unique peptides of the NrnA proteins and a low confidence match to a unique NrnB_*Bs*_ peptide.

Deletion of *nrnA* does not appear to affect growth in rich medium (Supplementary Figure S4); however, our in vitro data suggested that NrnA preferentially cleaves short RNAs. To investigate whether NrnA*_Bs_* might meaningfully affect these RNA substrates in vivo, we incubated a radiolabeled RNA with lysates extracted from *B. subtilis* cells grown to late-exponential phase (OD_600_ ∼1.0). Specifically, a 5′-radiolabeled 10-mer RNA was mixed with cell lysates that had been extracted from wild-type, Δ*nrnA*, or Δ*nrnB* strains and the products of these reactions were then resolved by 20% urea-denaturing PAGE (Figure 6A). This revealed that the 10-mer was readily degraded to nucleoside monophosphates by wild-type lysates. It also showed that *ΔnrnB* lysates generated a degradation profile that was identical to wild-type (Figure 6A). In contrast, there was specific accumulation of 2–4-mers when the 10-mer RNA was incubated with Δ*nrnA* lysates, concurrent with reduced levels of nucleoside monophosphates (Figure 6A). This observation suggests that NrnA*_Bs_* is the primary RNase responsible for cleaving 2–4-mers during late exponential phase and agrees well with our biochemical assessment of the purified proteins. We then integrated an inducible copy of *nrnA*_Bs_*, nrnB*_Bs_*, orn*_Vc_ or *cysQ_Ec_* into a nonessential locus of the Δ*nrnA* strain and prepared lysates for RNA degradation (Figure 6B). An empty vector control was included in this analysis. Δ*nrnA* complemented with an empty vector resembled the Δ*nrnA* strain, showing again an accumulation of 2–4-mers. In contrast, bands corresponding to 2- to 4-mers decreased upon complementation with *nrnA*, resembling lysates from the wild-type strain. Although Δ*nrnB* lysates did not show any change in degradation of the 10-mer RNA, complementation of the Δ*nrnA* strain with *nrnB* surprisingly resulted in complete processing of the RNA substrate, including all intermediates. Consistent with its role in processing dinucleotides, the complementation with *orn* led to a marked decrease in 2-mers, alongside a moderate decrease in 3-mers. However, complementation of Δ*nrnA* with *cysQ* showed the same RNA accumulation as Δ*nrnA*, confirming that CysQ does not play a role in RNA degradation.

To address whether in vitro hydrolysis of a deoxydinucleotide substrate NrnA*_Bs_* was possibly a physiologically relevant substrate for NrnA*_Bs_*, we conducted ex vivo experiments wherein a 5′-radiolabeled 20-mer DNA was incubated in cellular lysates extracted from wild-type, Δ*nrnA*, or a Δ*nrnA* strain containing an inducible copy of *nrnA* (Supplementary Figure S5). These data suggested that the wild-type lysate degraded the 20-mer DNA to near completion, resulting in accumulation of deoxymononucleotides. However, when we incubated the 20-mer DNA in the Δ*nrnA* cellular lysate, we observed an accumulation of deoxydinucleotides. In contrast, these deoxydinucleotides did not accumulate for the Δ*nrnA* strain expressing an inducible copy of *nrnA*. These data suggest that DNA dinucleotides may in theory represent viable substrates for NrnA*_Bs_*.

Since addition of the 10-mer RNA led to accumulation of short RNAs in lysates from the Δ*nrnA* mutant, we reasoned that this strain should also exhibit phenotypes that result from an overabundance of linear dinucleotides. Specifically, increased levels of dinucleotides are known to lead to an increase in c-di-NMPs (13,18,52). C-di-GMP abundance is decreased in *B. subtilis* motile cells (28); therefore, elevated dinucleotides could in theory alter the proportion of motile cells, by affecting c-di-GMP abundance. To test this, we inoculated the various complementation strains into soft agar and measured the radius of swimming motility in an endpoint assay (Figure 6C). This revealed that the Δ*nrnA* mutation led to a significant decrease in swimming motility, suggesting that this strain exhibits elevated dinucleotides (both linear and cyclic). Correspondingly, the complementation with *nrnA* fully restored swimming motility. A swimming defect was not observed in the Δ*nrnB* mutant (Figure 6C), suggesting that NrnA*_Bs_*is primarily responsible for maintaining cellular GG pools during exponential phase growth; however, complementation of the Δ*nrnA* strain with *nrnB* resulted in full restoration of swimming motility. Therefore, while NrnB is not required during exponential phase growth for degradation of short RNAs, our data suggests that it is proficient in this ability if forcibly expressed. Finally, complementation with *orn* only partially restored swimming motility, further hinting that the range of RNA substrates processed by NrnA might be broader than that of Orn, an enzyme that may specialize as a diribonuclease.

### NrnA*_Bs_* is expressed during exponential growth

To directly determine whether NrnA*_Bs_* or NrnB*_Bs_* are produced during exponential growth (OD_600_ = 0.8), an untargeted mass spectrometry proteomic analysis was performed on wild-type, Δ*nrnA* or Δ*nrnB* strains of *B. subtilis*. Peptide spectral matches (PSMs) associated with unique tryptic peptides from NrnA*_Bs_* versus the reference *B. subtilis* proteome were identified in the wild-type and Δ*nrnB* strains (Figure 6D and Supplementary Figure S6). A single PSM identified as a unique NrnB*_Bs_* peptide (Supplementary Figure S7) was found in the Δ*nrnA* strain. This match was determined to be a low confidence match by manually observation of the PSM as the identifying peaks are commiserate with peaks from instrument noise, thus it can be surmised that NrnB*_Bs_* could not be detected under these growth conditions. Therefore, we conclude that NrnA*_Bs_* is produced during vegetative growth, whereas NrnB*_Bs_* is not. Perhaps the latter is produced under different cellular conditions.

### NrnA*_Bs_* is widespread in Firmicutes

Our aggregate data have shown that, in our hands, purified NrnA*_Bs_* acts preferentially on short RNAs from two to four nucleotides in length and does not act on c-di-AMP or pAp. These biochemical observations are further bolstered by the nearly identical substrate preferences exhibited by Pde2*_Sp_* and NrnA*_Ef_*, suggesting that all three proteins might be members of a common subclass of standalone DHH-DHHA1 proteins. By extension, the differing substrate preferences of CnpB*_Mt_* suggests it represents a different subclass of DHH-DHHA1 proteins. However, it is not clear what sequence and structural features delineate these two subclasses of DHH-DHHA1 proteins. Nor is it clear whether yet more subclasses still await discovery. Therefore, the inability to correctly annotate DHH-DHHA1 proteins is a significant problem. Since our data showed that NrnA*_Bs_*, Pde2*_Sp_* and NrnA*_Ef_* closely resembled one another in their biochemical preferences, we sought to determine whether these proteins are members of a defined subclass of DHH-DHHA1 proteins.

The DHH domain of NrnA*_Bs_* and other DHH family proteins includes several distinct sequence motifs, each including a conserved aspartate residue (17). The C-terminal DHHA1 domain contains a GGGH-x-x-ASG motif that is likely to be adjacent to residues involved in substrate recognition. Also, an R-x-R-x-R motif (R262, 264 and 266 for NrnA*_Bs_*) may be conserved in NrnA-like proteins and has also been suggested to participate in substrate recognition (17,21). However, the range of residues and sequence motifs involved in selection of substrates has not yet been established. Nor has it been determined whether different subclasses of NrnA-like proteins may exhibit different substrate preferences and cellular purposes. Interestingly, a recent study demonstrated that a DHH-DHHA1 homolog from *Vibrio cholera* acts specifically on GG, at the exclusion of other dinucleotides and other short RNA species (55). This important publication demonstrates proof-of-principle that certain bacteria may have evolved specialized versions of DHH-DHHA1 proteins, and that still more cellular roles for DHH-DHHA1 proteins are likely to await discovery.

**Figure 7.**
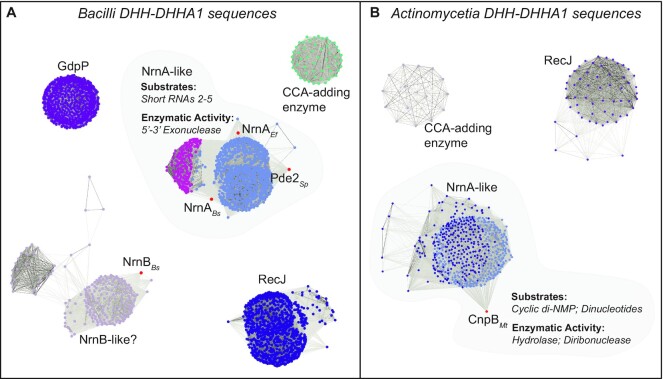
Sequence similarity network (SSNs) for DHH subfamily 1 proteins within selected taxonomical groups based on subsequences inclusive of only the DHH and DHHA1 domains. (A, B) Pairwise sequence similarities for DHH subfamily 1 proteins identified in two bacteria taxa (**A**) *Bacilli*, and (**B**) *Actinomycetia* are displayed in similarity networks, where nodes represent subsequences (concatenation of DHH and DHHA1 domain sequences) and edges are colored in grayscale to reflect the strength of the similarity, computed as –log_10_(*E*-value). The *E*-value was thresholded at (A) ≤10^−20^ and (B) ≤10^−15^; these cutoffs were manually adjusted to reveal substructures within clusters that likely indicate functionally relevant subsequence diversity. Clauset-Newman-Moore greedy modularity maximization was applied to partition the network, finding clusters of sequences exhibiting high, in-group subsequence similarity; isolate nodes and clusters which consisted of fewer than four members were removed from the graph. Different clusters are denoted by color, and clusters are annotated based on selected representatives.

Phylogenetic analysis of all proteins with a DHH-DHHA1 domain show that these domains can be standalone (‘NrnA-like’) or occur in tandem with other domains. For example, GdpP proteins, which specifically process c-di-AMP, utilize several domains alongside their DHH and DHHA1 domains. In contrast, the NrnA*_Bs_*, NrnA*_Ef_*, NrnA*_Sp_* and CnpB*_Mt_* proteins characterized herein do not have any recognizable domains other than the DHH and DHHA1 domains. As a preliminary test to determine if NrnA proteins could be identified by bioinformatics, we extracted the DHH and DHHA1 domain sequences of bacterial proteins from Bacilli and concatenated them together as a sub-sequence construct. Then, we performed a clustering analysis of these sequences with parameters that encouraged sequences to be closely clustered, even for pairs with large edit distances (Figure 7). This revealed several distinct clusters, including different groups of proteins corresponding to RecJ, CCA-adding enzymes, and GdpP. This analysis also revealed a bimodal cluster, one portion of which contained the sequences for NrnA*_Bs_*, NrnA*_Ef_*, and NrnA*_Sp_*. Given the similarities in their biochemical characteristics, as investigated herein, it is tempting to speculate that this overall collection of sequences (Supplementary Table S2) corresponds to a group of NrnA proteins that closely resembles NrnA*_Bs_*. Therefore, one would predict that these proteins process RNA substrates from 2- to 4-nucleotides in length, starting from their 5′ terminus. Notably, NrnB*_Bs_* was not found in the NrnA cluster of proteins. Instead, it could be identified within another cohesive group of related protein sequences, distinct from NrnA. From this, it is tempting to speculate that NrnB proteins may correspond to a specific class of DHH-DHHA1 proteins, functionally different from NrnA. As little is known about the proteins in this cluster, NrnB warrants further biochemical and structural investigation.

A clustering analysis of Actinobacteria DHH-DHHA1 sequences revealed a complex arrangement of overlapping clusters that contained CnpB*_Mt_*, CnpB*_Ms_*, CnpB*_Ma_* and CnpB*_Rr_*. From this, it is difficult to predict the overall diversity in cellular roles for these proteins, although it can be surmised that a large subset is likely to act specifically against c-di-NMP substrates. Without biochemically analyzing more proteins, the degree to which CnpB*_Mt_* is representative of these clusters of protein sequences is still unknown. Together, these data suggest that it may be possible to work toward prediction of NrnA proteins, at least from Bacilli, which share a core set of biochemical features. Yet these data also suggest that there are likely to be other groups of standalone DHH-DHHA1 proteins, including NrnB, that differ from NrnA in fundamental ways.

## DISCUSSION

Our experiments on purified NrnA*_Bs_* revealed a marked preference for cleavage of very short RNAs, from two to five nucleotides in length, which were specifically processed from their 5′ terminus. Moreover, our analysis showed that NrnA*_Bs_*preferentially hydrolyzes RNAs 2–4 nucleotides in length even when other longer RNA substrates are included in the same reaction. This observation was further bolstered by experiments wherein a radiolabeled oligonucleotide was added to cellular extracts and the RNase products were analyzed by denaturing electrophoresis. This showed that depletion of NrnA*_Bs_* resulted in accumulation of 2-mers, 3-mers and 4-mers, while, in contrast, deletion of NrnB*_Bs_* did not result in accumulation of these short RNAs under normal vegetative growth conditions. And while deletion of NrnA*_Bs_* led to accumulation of RNAs between 2 and 4 nucleotides in length, it also affected cellular signaling. Deletion of *nrnA* resulted in an increase in cyclic di-guanosine monophosphate (c-di-GMP) with a corresponding decrease in swimming motility. Therefore, the processing of dinucleotides by NrnA*_Bs_* is required for maintaining proper homeostasis of cyclic dinucleotide signaling molecules. We also purified NrnA-like proteins from several related species (*Streptococcus pyogenes* Pde2, *Enterococcus faecalis* NrnA, *Mycobacterium tuberculosis* CnpB) and assayed them using the same reaction conditions as with NrnA*_Bs_*. Pde2*_Sp_* and NrnA*_Ef_* exhibited a strong preference for short RNAs and closely resembled NrnA*_Bs_* overall. Moreover, our bioinformatic analysis of DHH-DHHA1 proteins showed that these proteins may be closely related to NrnA*_Bs_*. Therefore, together, our data demonstrate that a cohesive group of Firmicutes NrnA proteins are required for housekeeping processing of RNAs between two and four nucleotides in length. In contrast, CnpB-like proteins from *M. tuberculosis, M. smegmatis, M*. avium and *R. ruber* exhibited different substrate preferences when assayed under the same reaction conditions. While they were also capable of cleaving very short RNAs, the CnpB proteins were able to directly process c-di-AMP to NMPs, as compared to NrnA*_Bs_*, Pde2*_Sp_* and NrnA*_Ef_*, which were unable to cleave c-di-AMP under our assay conditions. Lastly, none of the proteins exhibited a significant level of pAp hydrolysis activity in vitro under our assay conditions. Interestingly, a recent biochemical analysis of the *Lysteria monocytogenes* NrnA showed virtually no activity when assayed for pAp hydrolysis (56). Therefore, our aggregate data show that NrnA proteins exhibit broader substrate preferences as compared to Orn. While Orn specifically processes dinucleotides, NrnA acts on RNAs between 2 and 4 nucleotides in length and processes them from their 5′ terminus. We also show that NrnA*_Bs_* is fully capable of processing DNA dinucleotides, although the physiological significance of this is still currently unclear and further work is needed to determine whether NrnA plays a meaningful role in processing cellular DNA dinucleotides.

NrnA*_Bs_* and NrnB*_Bs_*were previously assumed to be effectively synonymous in their biochemical activity and intracellular functions (14). In fact, NrnA*_Bs_* and NrnB*_Bs_* were initially identified based on their ability to complement a conditional *E. coli orn* mutant strain (14,15). Encoding for both NrnA and NrnB proteins does not appear to be a widespread arrangement amongst bacterial genomes and based on the data shown herein. NrnB*_Bs_* is not expressed during exponential growth, nor does it appear to process RNAs during exponential growth, in contrast to NrnA*_Bs_*. Therefore, we speculate that the presence of both an NrnA and NrnB protein has allowed *B. subtilis* the opportunity to specialize the functional role(s) of the latter protein. Interestingly, NrnB-like sequences appeared to cluster together into a separate but cohesive group of sequences; perhaps this indicates that they will share biochemical properties with each other, but distinct from NrnA. Therefore, in total, our studies argue that NrnA*_Bs_*is the housekeeping enzyme for degradation of short RNAs in *B. subtilis*, while NrnB*_Bs_*is likely to be expressed under a different phase of cellular growth. However, even with comprehensive analysis of *B. subtilis* NrnA, there are still gaps in our understanding of the degradation pathway for short RNAs. While deletion of *orn* genes is either lethal or results in severe growth defects for the gammaproteobacterial species in which it has been mutated, deletion of *nrnA* does not present *B. subtilis* with a severe growth defect (Supplementary Figure S4). This may suggest that there is redundancy in this step of the RNA degradation pathway or that another RNase can accommodate removal of dinucleotides upon depletion of NrnA, assuming that accrual of dinucleotides is the basis of the growth phenotype observed for *orn* mutant strains. This once again highlights how the RNA degradation pathway differs dramatically between *E. coli* and *B. subtilis*. And as a corollary to that statement, the complexity and variation between the suite of *E. coli* and *B. subtilis* RNases is likely to broaden even further when other, non-model bacteria are considered.

Standalone DHH-DHHA1 domain containing proteins are broadly distributed amongst many taxonomic groups of bacteria, and the members of this protein family are commonly annotated as either NrnA or NrnB proteins (12). A great challenge going forward will be to annotate appropriately the specialized members of this protein family. There have been multiple reports suggesting that NrnA-like proteins are functionally diverse, although some of these prior published data have been debated extensively. For example, in a biochemical analysis of the NrnA-like proteins from *T. maritima* and *S. pneumoniae*, it was surmised that previously reported c-di-AMP hydrolysis activity of the *S. pneumoniae* and *M. tuberculosis* NrnA-like proteins might be an artifact of assay conditions (49). Yet in another analysis, it was suggested that the *S. aureus* NrnA-like protein preferred cleavage of the dinucleotide AA, but that c-di-AMP could also be an intracellular substrate for this protein (18). While our data agrees that a large subset of NrnA-like proteins is unlikely to hydrolyze c-di-AMP, our biochemical assays suggested that c-di-AMP is indeed a primary substrate for the *M. tuberculosis, M. smegmatis, M. avium* and *R. ruber* CnpB proteins. It is therefore tempting to speculate that c-di-AMP hydrolysis activity is the primary function of the NrnA-like/CnpB proteins encoded by many actinobacterial organisms. In contrast, our sequence clustering analysis suggests that the NrnA-like proteins found in Bacilli function primarily to hydrolyze 2–4 mer RNAs. The current annotation convention for the standalone DHH-DHHA1 protein family will undoubtedly need to be updated to account for the functionally distinct members of this protein family. Indeed, this will be exceedingly difficult in the absence of characteristic sequence motifs that predict the enzyme's substrate preference(s). However, rigorous enzymological analysis will also be essential in determining the substrate specificity of these enzymes. In the study herein, we find great value in directly comparing the biochemical properties of different purified enzymes under a common set of reaction conditions. In future experiments, these efforts can be expanded for examination of many other proteins to improve the functional annotation of different subclasses of DHH-DHHA1 protein sequences.

## DATA AVAILABILITY

Mendeley Data is an open-source depository of primary data (https://data.mendeley.com/my-data/). Upon publication, all data will be accessible with a public accession number (doi: 10.17632/rtkt5t6yt9.1).

## Supplementary Material

gkac1091_Supplemental_File

## Notes

Present address: Cordelia A. Weiss, Section of Molecular Microbiology and Medical Research Council Centre for Molecular Bacteriology and Infection, Imperial College London, London SW7 2AZ, UK.
