# Supplementary material for: NrnA is a 5′-3′ exonuclease that processes short RNA substrates in vivo and in vitro
Source: Nucleic Acids Res. 2022 Dec 7;50(21):12369–88. doi: 10.1093/nar/gkac1091 (PMC9757072; doi:10.1093/nar/gkac1091)
Supplement: gkac1091_Supplemental_File [file gkac1091_supplemental_file.pdf]

NrnA is a 5'-3' Exonuclease that Processes Short RNA Substrates *In Vivo* and *In Vitro*

Cordelia A. Weiss[1]†, Tanner M. Myers[2]†, Chih Hao Wu[1], Conor Jenkins[2], Holger Sondermann[3,4], Vincent T. Lee[1], Wade C. Winkler[1,2]*
[1] Department of Cell Biology and Molecular Genetics, University of Maryland, College Park, Maryland, 20742, USA
[2]Department of Chemistry and Biochemistry, University of Maryland, College Park, Maryland, 20742, USA
[3]Centre for Structural Systems Biology, Deutsches Elektronen-Synchrotron (DESY), 22607 Hamburg, Germany
[4]Christian-Albrechts-Universität, 24118 Kiel, Germany

†Joint Authors
* To whom correspondence should be addressed. Tel: (301) 405-7780; Email: wwinkler@umd.edu
Present Address: Cordelia A. Weiss, Section of Molecular Microbiology and Medical Research Council Centre for Molecular Bacteriology and Infection, Imperial College London, London, SW72AZ, United Kingdom

CONTENTS

Page		
2	Figure S1.	Purification of enzymes used in this study
3	Figure S2.	Cleavage assay of NrnA*Bs* with 5 nM RNA substrates
4	Figure S3.	Cleavage assay of CnpB*Mt* with RNA substrates
5	Figure S4.	Growth curve of wild-type, Δ*nrnA* and Δ*nrnB*
6	Figure S5.	*Ex vivo* cleavage of 20-mer DNA substrate
7	Figure S6.	Mass spectrometry: Peptide spectrum of NrnA*Bs*
8	Figure S7.	Mass spectrometry: Peptide spectrum of NrnB*Bs*
9	Table S1.	Strains list
10	Table S2.	DHH-DHHA1 protein sequences that cluster with NrnA*Bs*, NrnA*Ef*, and Pde2*Sp*

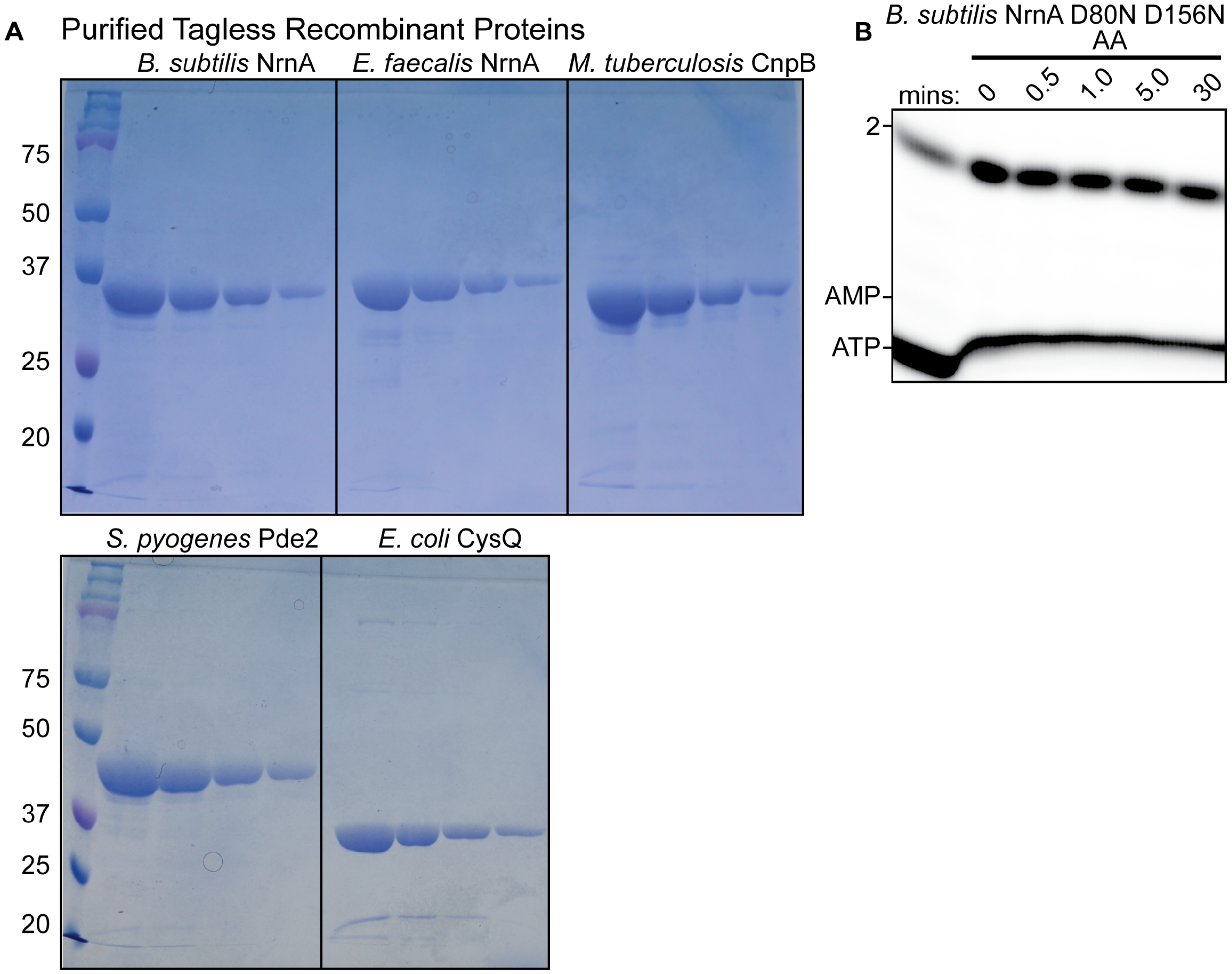

Figure S1. Assessment of protein purity and inactivation of the NrnA$_{Bs}$ active site mutant. (A) SDS-PAGE analysis of proteins analyzed in this study. (B) 100 nM of NrnA$_{Bs}$ D80N D156N incubated with 1 µM of dinucleotide substrate. Aliquots were removed from the reactions and quenched in 150 mM EDTA and 4 M Urea. Analysis of the lack of degradation products proceeded by denaturing PAGE.

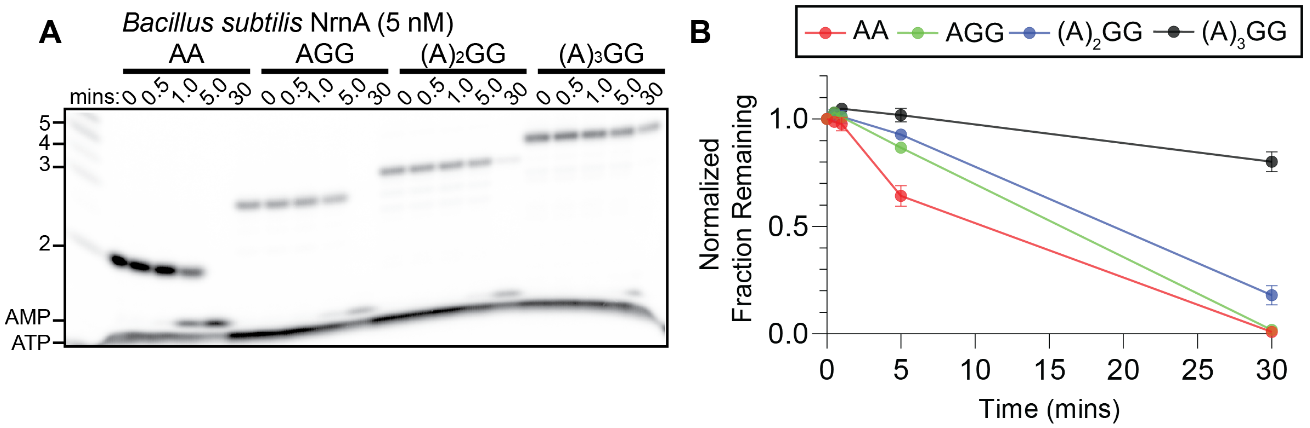

Figure S2. *B. subtilis* NrnA displays a slight dinucleotide preference under rate limiting conditions. (A) Native RNA molecules 2 – 5 nucleotides in length at a final concentration of 1 µM containing trace amounts of ^{32}P radiolabeled RNA were subjected to a time course cleavage reaction by 5 nM of purified $NrnA_{Bs}$. Aliquots were removed from reactions and quenched in 150 mM EDTA and 4 M urea. Degradation products were resolved by denaturing PAGE. (B) Quantification of the normalized radioactive intensity of the initial substrate depletion over time as the average and SD of 3 independent experiments in (A).

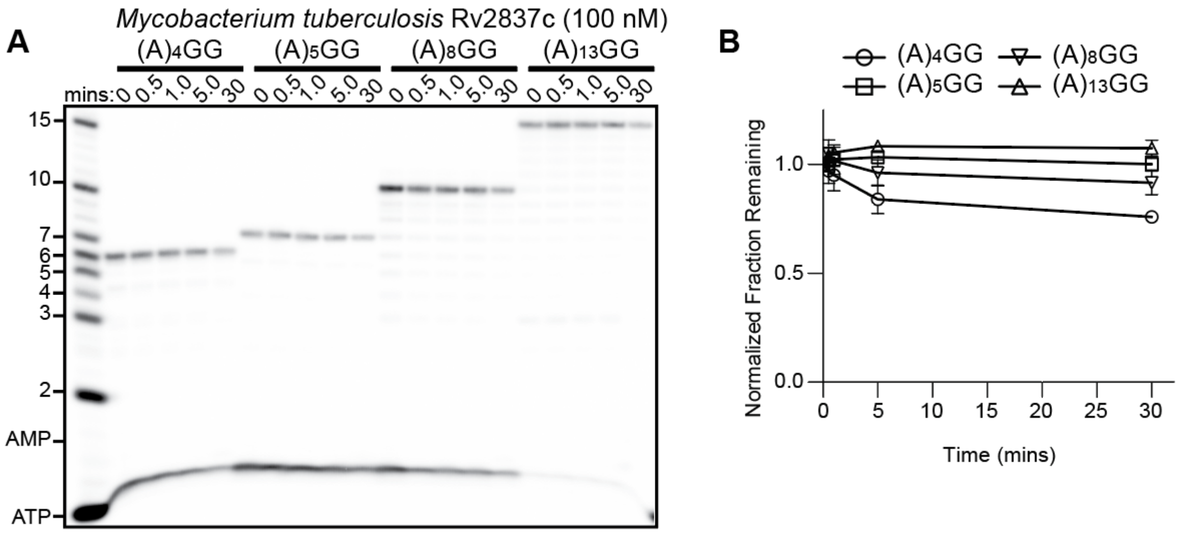

Figure S3. CnpB*Mt* (Rv2837c) does not appreciably cleave longer RNA substrates. (A) Native RNA molecules 6, 7, 10, and 15 nucleotides in length at a final concentration of 1 μM containing trace amounts of ^{32}P radiolabeled RNA were subjected to a time course cleavage by 100 nM of CnpB*Mt*. Aliquots were removed from reactions and quenched in 150 mM EDTA and 4 M urea. The degradation products were resolved by denaturing PAGE. (B) Quantification of the normalized radioactive intensity of the initial substrate depletion over time plotted as the average and SD of 3 independent experiments.

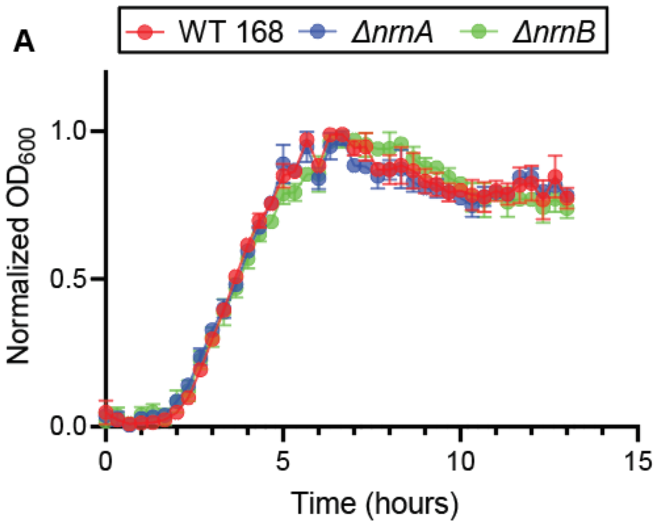

Figure S4. Growth curve of wild type, ∆*nrnA* and ∆*nrnB*. (A) Overnight cultures of respective strains of *B. subtilis* were back diluted to an OD_{600} of 0.1 and left to grow in a SPECTROstar® nano shaking plate reader at 37°C. Quantification of the normalized OD_{600} over time plotted as the average and SD of 3 independent experiments.

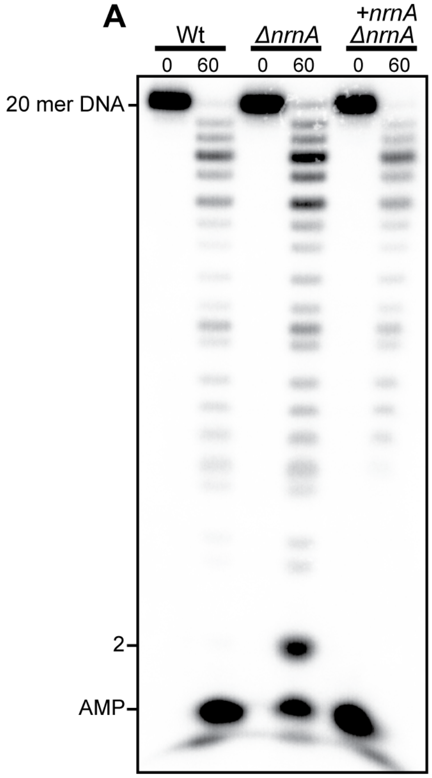

Figure S5. NrnA$_{Bs}$ degrades deoxydinucleotides in *B. subtilis* cellular lysates. (A) Whole cell lysates of WT *B. subtilis, ΔnrnA*, or *ΔnrnA* complementation strains containing IPTG inducible expression of *nrnA$_{Bs}$* were harvested during vegetative growth and incubated with 1 μM of a 5' radiolabeled 20mer DNA. DNA degradation products were resolved by denaturing PAGE.

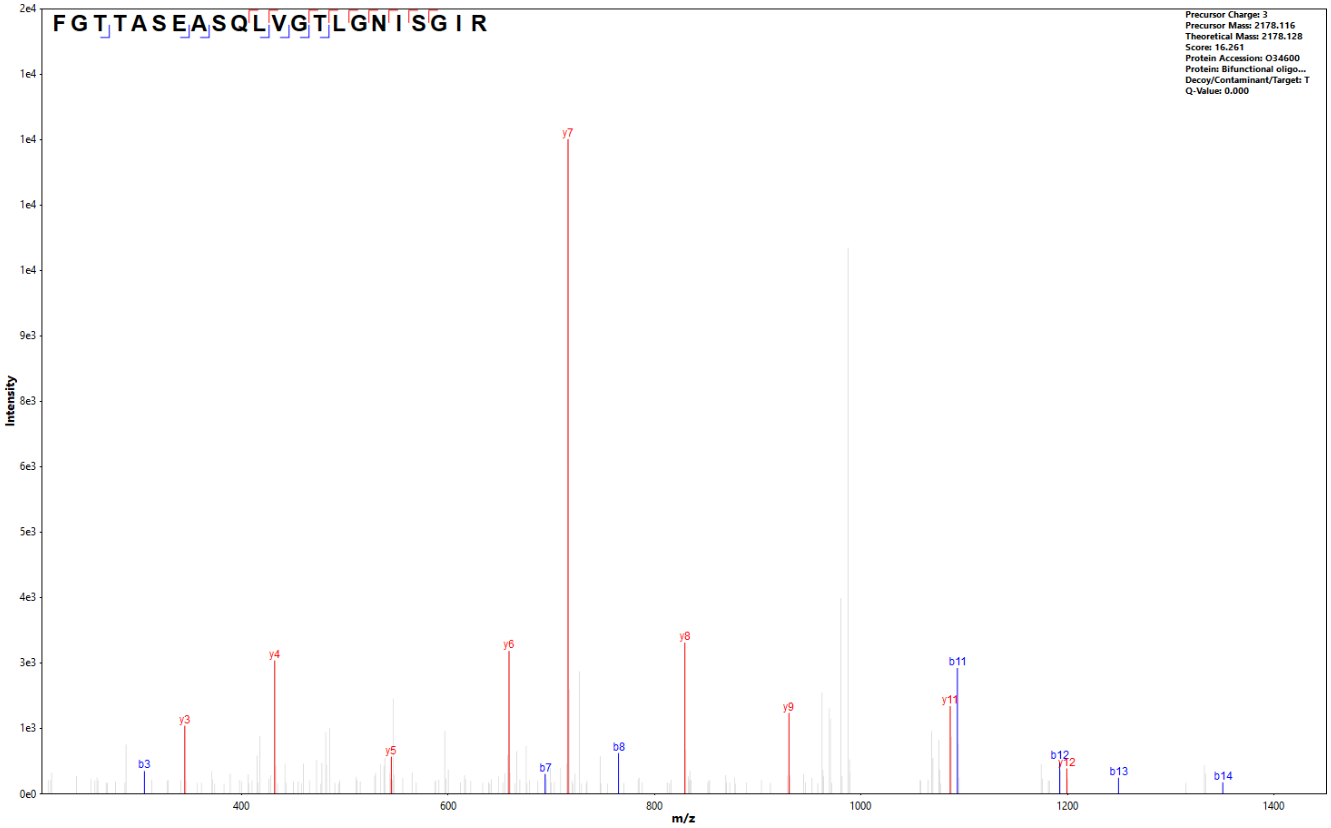

Figure S6. A selected fragmentation spectra assigned to a unique NrnA$_{Bs}$ peptide identified in wild-type *B. subtilis*. The signal from the fragmented peptide is well above the background signal intensity.

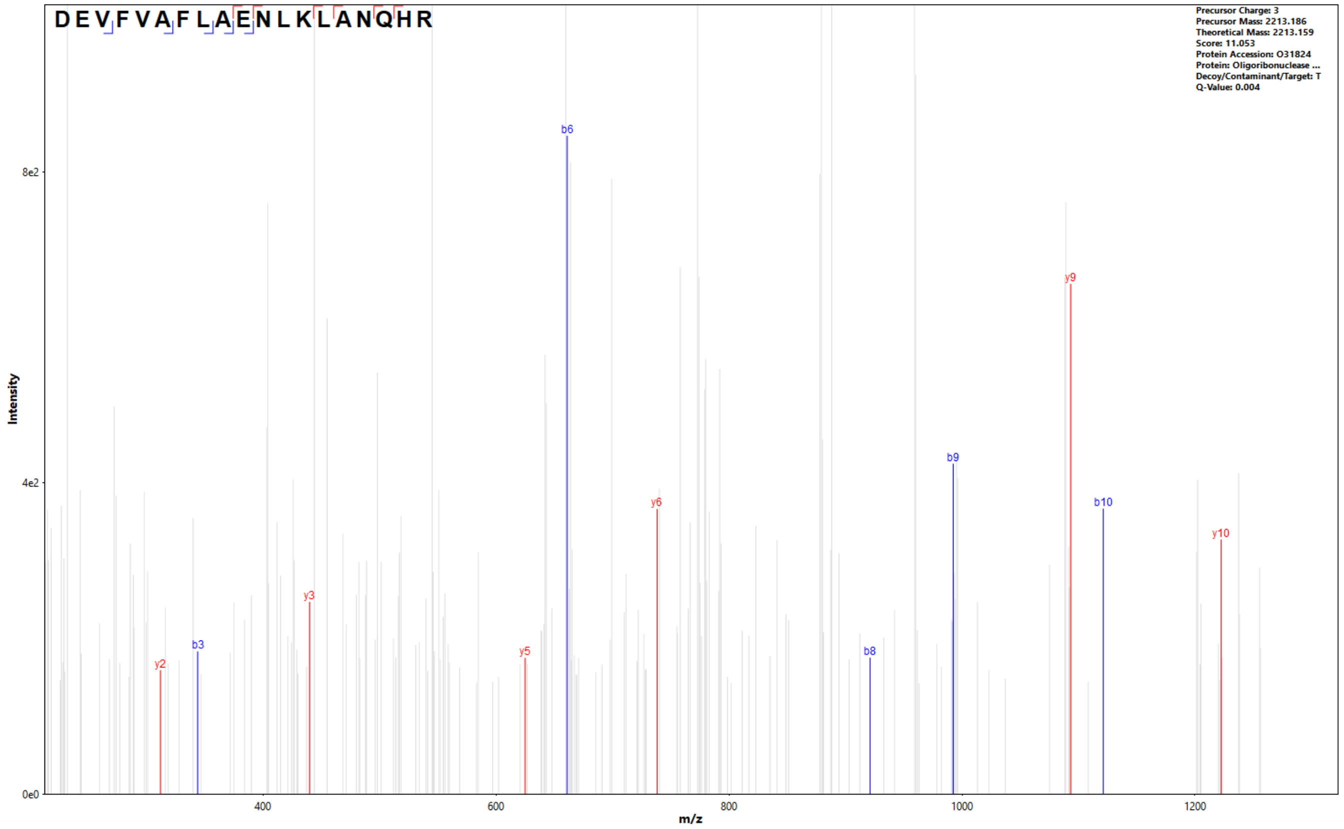

Figure S7. A selected fragmentation spectra assigned to a single unique NrnB$_{Bs}$ peptide identified in the Δ*nrnA B. subtilis* strain. The signal from the fragmented peptide is low, with the dominant peak being ~8e^{2}. This intensity corresponds to a signal which is essentially indistinguishable when compared to the background.

Table S1.

Strain Name	Genotype	Antibiotic	Source
***B. subtilis* Strains**[a]			
168	WT, *trpC2*		BGSC[b]
Weiss104	Δ*nrnB*		BGSC[b]
Weiss106	Δ*nrnA*		BGSC[b]
JG040	*amyE*::P_{const}-*yfp cat*	Chloramphenicol	(Weiss et al., 2019)
Weiss424	Δ*nrnB amyE*::P_{const}-*yfp cat*	Chloramphenicol	This study
Weiss425	Δ*nrnA amyE*::P_{const}-*yfp cat*	Chloramphenicol	This study
RSL_F4	*amyE*::P_{const}-*lchAA* leader-*yfp cat*	Chloramphenicol	(Weiss et al., 2019)
Weiss428	Δ*nrnB amyE*::P_{const}-*lchAA* leader-*yfp cat*	Chloramphenicol	This study
Weiss429	Δ*nrnA amyE*::P_{const}-*lchAA* leader-*yfp cat*	Chloramphenicol	This study
Weiss520	Δ*nrnA amyE*::P_{hs}-$nrnA_{Bs}$ *spec*	Spectinomycin	This study
Weiss521	Δ*nrnA amyE*::P_{hs}-$nrnB_{Bs}$ *spec*	Spectinomycin	This study
Weiss522	Δ*nrnA amyE*::P_{hs}-orn_{Vc} *spec*	Spectinomycin	This study
Weiss523	Δ*nrnA amyE*::P_{hs}-$cysQ_{Ec}$ *spec*	Spectinomycin	This study
Weiss524	Δ*nrnA amyE*::P_{hs} *spec*	Spectinomycin	This study
Weiss536	*amyE*::P_{hs} *spec*	Spectinomycin	This study
***E. coli* Strains and Plasmids**[c]			
Weiss449 (pWeiss151)	10xHis-SUMO-$NrnA_{Ef}$	Carbenicillin	This study
Weiss451 (pWeiss152)	10xHis-SUMO-$NrnA_{Bs}$	Carbenicillin	This study
Weiss453 (pWeiss153)	10xHis-SUMO-$Pde2_{Sp}$	Carbenicillin	This study
Weiss457 (pWeiss155)	10xHis-SUMO-$CnpB_{Mt}$	Carbenicillin	This study
Weiss468 (pWeiss164)	10xHis-SUMO-$CysQ_{Ec}$	Carbenicillin	This study
Weiss500 (pWeiss190)	10xHis-SUMO-$DisA_{Bt}$	Carbenicillin	This study
TMM003 (pTMM2)	10xHis-SUMO-NrnA $(D80N\ D156N)_{Bs}$	Carbenicillin	This study
TMM076 (pTMM38)	6xHis-$PPDK_{Cs}$	Carbenicillin	This study
TMM096 (pTMM48)	10xHis-SUMO-Orn_{Ec}	Carbenicillin	This study
TMM156 (pTMM72)	10xHis-SUMO-$CnpB_{Ma}$	Carbenicillin	This study
TMM158 (pTMM73)	10xHis-SUMO-$CnpB_{Ms}$	Carbenicillin	This study
TMM162 (pTMM75)	10xHis-SUMO-$CnpB_{Rr}$	Carbenicillin	This study

[a]All *B. subtilis* strains are derivatives of 168 unless otherwise noted
[b]Bacillus Genetic Stock Center
[c]All *E. coli* strains are derivatives of T7 Express unless otherwise noted

Table S2. DHH-DHHA1 protein sequences that cluster with $NrnA_{Bs}$, $NrnA_{Ef}$, and $Pde2_{Sp}$.

Gene names	Organism	Length
CLV38_12416	Alkalibacterium olivapovliticus	326
Tpal_394	Trichococcus palustris	321
HMPREF2904_04550	Streptococcus sp. HMSC072G04	314
BLX88_18365	Bacillus obstructivus	311
FD19_GL001705	Lactobacillus thailandensis DSM 22698 = JCM 13996	361
nrnA BDKNPLJD_01216 DM475_05695	Lactobacillus helveticus (Lactobacillus suntoryeus)	318
T233_01514	Vagococcus lutrae LBD1	315
GH754_15510	Salinibacillus xinjiangensis	318
FAM10859_02576	Lactobacillus paracasei	310
Eab7_2064	Exiguobacterium antarcticum (strain B7)	312
ATW55_00940	Acidibacillus ferrooxidans	340
SD77_1092	Bacillus badius	313
ERX40_04975	Macrococcus carouselicus	313
D1970_16690	Mesobacillus zeae	316
HMPREF0555_1030	Leuconostoc mesenteroides subsp. cremoris ATCC 19254	326
FD46_GL001218	Lactobacillus oeni DSM 19972	319
GFC29_2031	Anoxybacillus sp. B7M1	315
D7Z54_06940	Bacillus salarius	316
CJ229_04235	Nosocomiicoccus massiliensis	319
D1010_17345 FMM01_12010	Schleiferilactobacillus harbinensis	316
UKC_03394	Enterococcus gilvus ATCC BAA-350	317
SAMN05216238_102392	Lentibacillus persicus	315
B0X71_06720	Planococcus sp. Y42	316
Q783_03125	Carnobacterium inhibens subsp. gilichinskyi	316
C7J90_10055	Staphylococcus felis	313
ytqI HBHAL_3771	Halobacillus halophilus (strain ATCC 35676 / DSM 2266 / JCM 20832 / KCTC 3685 / LMG 17431 / NBRC 102448 / NCIMB 2269) (Sporosarcina halophila)	315
FYJ81_00810	Staphylococcus sp. McC-251-APC-3A2	311
STRDD10_00168	Streptococcus sp. DD10	312
JG30_04160	Bombilactobacillus mellifer	317
FD16_GL000025	Lactobacillus suebicus DSM 5007 = KCTC 3549	319
SAMN05877753_105185	Bacillus oleivorans	320
FRX57_02180	Streptococcus pharyngis	318
F5ESL0246_05895	Lactobacillus sp. ESL0246	318
CYJ29_08120 DBT54_03555	Aerococcus urinae	321
AB986_16265	Alkalihalobacillus macyae	311
FFIC_280390	Fructobacillus ficulneus	322
FN924_12030	Radiobacillus deserti	314
GMD78_00410	Ornithinibacillus caprae	314
DLJ74_06660	Gracilibacillus dipsosauri	312

OKIT_1151	Oenococcus kitaharae DSM 17330	315
nrnA OSO01_28290	Oceanobacillus sojae	314
I573_01948	Enterococcus sulfureus ATCC 49903	318
I568_01719	Enterococcus columbae DSM 7374 = ATCC 51263	312
SAMN05518683_11630	Salibacterium halotolerans	317
GS18_0201425	Metabacillus indicus (Bacillus indicus)	309
M670_01358	Bacillus azotoformans MEV2011	315
SRT_07610	Streptococcus troglodytae	310
SAG0537	Streptococcus agalactiae serotype V (strain ATCC BAA-611 / 2603 V/R)	311
HLI_17910	Halobacillus litoralis	318
GL993_06630	Facklamia sp. 252	327
EC501_04520	Lysinibacillus halotolerans	314
CD134_03160	Staphylococcus lutrae	310
D1B33_15665	Lysinibacillus yapensis	317
B0H94_109147	Salsuginibacillus halophilus	318
FC72_GL000735	Lactobacillus tucceti DSM 20183	317
STRUR_0776	Streptococcus urinalis 2285-97	314
AFL42_04845	Oceanobacillus caeni	317
SAMN04488053_10241	Alkalicoccus daliensis	313
BLX87_04545	Bacillus sp. VT-16-64	312
CKF48_16415	Cytobacillus kochii	314
FC36_GL000350	Lactobacillus equi DSM 15833 = JCM 10991	327
E4663_03405	Halobacillus salinus	315
D1B32_07745	Oceanobacillus profundus	315
HMPREF1208_01069	Staphylococcus sp. HGB0015	310
ABM34_04845	Companilactobacillus ginsenosidimutans	318
IV53_GL001008	Lactobacillus ceti DSM 22408	317
ASS94_12565	Staphylococcus equorum	311
E4T68_01760	Granulicatella sp. WM01	319
FYJ62_04505	Lactobacillus porci	321
FYJ82_00700	Streptococcus alactolyticus	315
DFP99_0121 WSWS_00492	Weissella soli	315
FC69_GL000429	Lactobacillus fuchuensis DSM 14340 = JCM 11249	318
FQV30_11300	Planomicrobium sp. CPCC 101110	312
QI30_01645	Kurthia sp. 3B1D	309
EDM58_15525	Brevibacillus panacihumi	331
BSQ49_03665	Liquorilactobacillus hordei	316
HMPREF9257_0986	Eremococcus coleocola ACS-139-V-Col8	325
EJA10_07970	Mesobacillus subterraneus	311
AB432_017810	Brevibacillus brevis (Bacillus brevis)	331
ERX55_02455	Macrococcus bovicus	314
SAMN05216498_1855	Tenuibacillus multivorans	318

EDD68_10443	Melghiribacillus thermohalophilus	320
BW732_04145	Vagococcus penaei	315
B2M26_14115	Acidibacillus ferrooxidans	340
OXB_3277	Bacillus sp. (strain OxB-1)	311
AEQ18_03895	Enterococcus sp. RIT-PI-f	314
FS935_14185	Metabacillus litoralis	312
SAMN05878482_101465	Peribacillus simplex	312
AC625_18240	Peribacillus loiseleuriae	313
ETI04_03520	Macrococcus canis	314
BEN83_06290 CYR79_01330	Ligilactobacillus agilis	318
CAI16_17610	Virgibacillus dokdonensis	315
FN960_00370	Bacillus sp. P16(2019)	311
BU064_01515	Staphylococcus succinus	311
DT065_07870	Salicibibacter kimchii	315
C7437_10166	Psychrobacillus insolitus	309
SAMN05192533_102387	Mesobacillus persicus	310
D8M04_00930	Oceanobacillus piezotolerans	313
SAMN04488559_10527	Isobaculum melis	317
GH885_02650	Gracilibacillus thailandensis	314
nrnA NCTC11323_00446	Gemella morbillorum	327
nrnA BCO26_1975	Bacillus coagulans (strain 2-6)	312
nrnA EXIGUO8A_11065	Exiguobacterium sp. 8A	322
D3X11_01815	Streptococcus sp. X16XC17	314
EK386_01970	Lysinibacillus antri	314
D822_08440	Streptococcus ratti FA-1 = DSM 20564	310
CL176_07760	Suicoccus acidiformans	329
SAMN04487944_11735	Gracilibacillus ureilyticus	313
E4T67_01075	Gemella sp. WT2a	330
SAMN02910293_02258	Streptococcus henryi	311
SAMN04488700_2342	Carnobacterium iners	319
BSQ50_03000	Liquorilactobacillus nagelii	319
P746_01675	Enterococcus faecalis CBRD01	317
LFYK43_10260	Ligilactobacillus salitolerans	321
D4T97_006245	Bacillus acidinfaciens	312
BIV59_13050	Bacillus sp. MUM 13	312
FB479_101692	Brevibacillus sp. AG162	331
RU97_GL001633	Enterococcus canis	315
CVD19_05880	Bacillus sp. T33-2	310
BSOLF_0118	Candidatus Carbobacillus altaicus	342
B4121_0875	Bacillus paralicheniformis	325
SAMN04488072_11194	Lentibacillus halodurans	315
CJ195_13410	Bacillus sp. UMB0899	313

E1I69_02705	Bacillus timonensis	313
nrnA_2 D8846_02490	Streptococcus oralis	314
HMPREF3215_02558	Staphylococcus simulans	312
HMPREF0444_0472	Granulicatella adiacens ATCC 49175	321
CYJ57_02250	Facklamia ignava	315
FC18_GL002135	Lactobacillus sharpeae JCM 1186 = DSM 20505	315
GRB29_00875	Streptococcus pneumoniae	313
nrnA L248_1855	Lactobacillus shenzhenensis LY-73	313
FC43_GL001032	Lactobacillus ingluviei DSM 15946	236
nrnA_1 BkAM31D_18730	Alkalihalobacillus krulwichiae	309
E2L07_00610	Alkalihalobacillus halodurans (Bacillus halodurans)	314
ERX29_06070	Macrococcus lamae	313
ABE28_006360	Peribacillus muralis	312
C8K15_10487	Paenisporosarcina sp. OV554	311
J416_08192	Gracilibacillus halophilus YIM-C55.5	313
OMQ_02086	Enterococcus saccharolyticus subsp. saccharolyticus ATCC 43076	318
COJ96_14380	Bacillus sp. AFS073361	316
LA20249_00090	Lactobacillus alimentarius DSM 20249	318
HMPREF1871_00668	Gemella asaccharolytica	327
C6W54_09930	Bacillaceae bacterium	312
C5L23_001019	Leuconostoc fallax	317
AS888_07390	Peribacillus simplex	312
CH76_02385	Lysinibacillus sp. BF-4	311
FC96_GL000638	Lactobacillus kimchicus JCM 15530	320
FC86_GL000297	Holzapfelia floricola DSM 23037 = JCM 16512	318
AKG37_03670	Bacillus australimaris	313
CEQ21_17770	Bacillus circulans	311
B1NLA3E_17220	Bacillus sp. 1NLA3E	307
B0I26_103207	Anoxybacillus vitaminiphilus	314
LCK_01243	Leuconostoc citreum (strain KM20)	336
FLP15_06535	Lactococcus sp. KACC 19320	308
IV45_GL001148	Limosilactobacillus secaliphilus	316
SAMN05192557_0622	Aliicoccus persicus	324
FD01_GL000338	Lactobacillus manihotivorans DSM 13343 = JCM 12514	316
EDD62_0348	Abyssicoccus albus	318
SAMN05443094_101787	Domibacillus enclensis	312
DCC39_09805	Pueribacillus theae	317
EII38_08425	Streptococcus minor	323
BN609_00259	Staphylococcus sp. CAG:324	332
ETI08_06125	Macrococcus goetzii	314
BM613_05535	Acidibacillus sulfuroxidans	343
DFR57_10921	Saliterribacillus persicus	310

SAMN05421670_2150	Psychrobacillus psychrotolerans	309
BN53_04015	Lactobacillus pasteurii DSM 23907 = CRBIP 24.76	319
E5351_05075	Lactobacillus intestinalis	317
CD29_14665	Lysinibacillus manganicus DSM 26584	314
GCWU000182_000264	Abiotrophia defectiva ATCC 49176	328
ELQ35_20205	Peribacillus cavernae	310
B7705_03105	Streptococcus oralis subsp. dentisani	311
AX762_06060	Alkalibacterium sp. 20	321
WQ54_12880 WQ54_17720	Bacillus sp. SA1-12	314
A5880_002697	Enterococcus sp. 4G2_DIV0659	316
D7I45_04385	Apilactobacillus bombintestini	327
HMPREF9024_01153	Pediococcus acidilactici 7_4	311
CD122_02020	Staphylococcus rostri	311
FPQ13_04260	Allobacillus sp. SKP4-8	320
GQ671_03915	Salinicoccus hispanicus	320
nrnA_2 FILTAD_01494	Filibacter tadaridae	313
AKK44_03120	Streptococcus phocae	316
nrnA CD116_09060 NCTC13712_01693	Staphylococcus schweitzeri	313
FD09_GL003152	Lactobacillus perolens DSM 12744	320
B8A42_06675	Dolosigranulum pigrum	322
F4V44_01245	Bacillus endozanthoxylicus	315
BVJ53_00020	Lacticaseibacillus chiayiensis	310
MUDAN_MDHGFNIF_02364	Lactiplantibacillus mudanjiangensis	309
FD27_GL001584	Lactobacillus frumenti DSM 13145	317
P343_02080	Sporolactobacillus laevolacticus DSM 442	309
SAMN02745189_00202	Salinicoccus alkaliphilus DSM 16010	323
FVP42_01120	Lactococcus sp. dk310	307
JCM19055_2373	Geomicrobium sp. JCM 19055	318
FC83_GL000328	Agrilactobacillus composti DSM 18527 = JCM 14202	321
FC21_GL000026	Lactobacillus equigenerosi DSM 18793 = JCM 14505	315
BKP56_07370	Marinilactibacillus sp. 15R	322
FD03_GL000545	Lactobacillus nodensis DSM 19682 = JCM 14932 = NBRC 107160	318
DCE79_13360	Lysinibacillus sp. 2017	314
BTR25_02455	Bacillus sp. MRMR6	312
FAX13_03240	Ligilactobacillus animalis	316
EPH95_08160	Salicibibacter halophilus	315
A6E74_04160	Enterococcus thailandicus	323
BU590_04800	Staphylococcus agnetis	311
CIB95_06310	Lottiidibacillus patelloidae	312
SAMN05421503_3445	Terribacillus aidingensis	313
DOE78_18520	Bacillus sp. Y1	310
nrnA DJ93_1624	Bacillus clarus	310

SAMN05216565_101137	Litchfieldia salsus	311
FOI68_07690	Brevibacillus sp. LEMMJ03	331
FC34_GL001657	Lactobacillus brantae DSM 23927	322
PS3_10195	Lactobacillus gastricus PS3	319
FPQ10_04370	Allobacillus sp. SKP2-8	320
DES48_10463	Paraliobacillus ryukyuensis	314
EBB45_03045	Lysinibacillus composti	314
nrnA_2 BN1058_02470	Paraliobacillus sp. PM-2	314
FD14_GL001970	Lactobacillus similis DSM 23365 = JCM 2765	319
GI482_03960	Bacillus sp. N3536	310
WQ57_09155	Mesobacillus campisalis	310
A5888_001082	Enterococcus sp. 9E7_DIV0242	320
A5869_000333	Enterococcus cecorum	319
E2R51_01010	Jeotgalibacillus sp. S-D1	310
SK667_1449	Streptococcus mitis	311
N783_14285	Pontibacillus marinus BH030004 = DSM 16465	316
CBF31_06495	Vagococcus fessus	315
nrnA NCTC10815_00705	Listeria grayi (Listeria murrayi)	313
HMPREF3291_16005	Bacillus sp. HMSC76G11	311
LA20249_08625	Lactobacillus alimentarius DSM 20249	314
nrnA_1 BN988_00075	Oceanobacillus picturae	314
CWR45_01495	Oceanobacillus chungangensis	313
SGA02_12240	Staphylococcus gallinarum	311
GH741_09230	Aquibacillus halophilus	315
SAMN05421767_11213	Granulicatella balaenopterae	318
A0126_10915	Exiguobacterium sp. N4-1P	312
SAMN02745116_02187	Pilibacter termitis	311
nrnA_2 JEOSCH030_01427	Jeotgalicoccus schoeneichii	319
SAMN04488127_1615	Bhargavaea ginsengi	311
DYJ42_06095 RN79_04965 SCODD09_00180	Streptococcus constellatus	312
FHP05_11810	Cerasibacillus terrae	319
JCM19046_351	Bacillus sp. JCM 19046	319
D7Z54_13095	Bacillus salarius	316
GS3922_02480	Geobacillus subterraneus	315
CHH53_16795	Terribacillus sp. 7520-G	313
nrnA_2 BN983_02938	Halobacillus karajensis	319
C5L31_000905	Secundilactobacillus malefermentans	319
ESZ50_09565	Weissella muntiaci	314
CAC02_05235	Streptococcus gallolyticus	314
CD039_05490	Staphylococcus argensis	311
DWV91_09525	Enterococcus asini	322
DIY07_06340	Streptococcus iniae (Streptococcus shiloi)	313

SAMN05878443_1109	Carnobacterium alterfunditum	316
JMA_24350	Jeotgalibacillus malaysiensis	311
WKK_00780	Weissella koreensis (strain KACC 15510)	310
CJ218_07355	Gemella sanguinis	325
DRW41_14375	Bacillus piezotolerans	310
FC84_GL000892	Lapidilactobacillus dextrinicus DSM 20335	321
C7K43_10975	Tetragenococcus koreensis	318
SAMN04488098_10272	Alkalibacterium thalassium	325
SAMN05428981_1011000	Bacillus sp. OV194	316
IV52_GL000571	Lactobacillus lindneri DSM 20690 = JCM 11027	318
CEW92_04690	Bacillaceae bacterium SAS-127	314
SAMN04488577_1498	Bacillus sp. cl95	310
CWS01_03720	Bacillus nealsonii	311
AJ85_18935 BALCAV_0221235	Bacillus alcalophilus ATCC 27647 = CGMCC 1.3604	318
CHH83_12130	Bacillus sp. 7586-K	312
BleG1_2720	Bacillus lehensis G1	309
JCM9140_4296	Bacillus wakoensis JCM 9140	311
CON00_22375	Bacillus sp. AFS096315	312
nrnA SAMEA4384403_01048	Mammaliicoccus stepanovicii	308
DS831_00190	Bombilactobacillus bombi	315
IV68_GL000498	Weissella halotolerans DSM 20190	314
PMI08_04885	Brevibacillus sp. CF112	331
HW35_05730	Bacillus sp. X1(2014)	310
nrnA_2 NCTC13832_01053 TP70_10375	Staphylococcus microti	311
nrnA_2 JEOPIN946_00937	Jeotgalicoccus pinnipedialis	319
Bsel_1343	Bacillus selenitireducens (strain ATCC 700615 / DSM 15326 / MLS10)	314
X560_0512	Listeria fleischmannii 1991	313
FC75_GL001683	Lactobacillus camelliae DSM 22697 = JCM 13995	324
EJN90_11695	Jeotgalibaca ciconiae	325
LrDSM24759_02280	Lactobacillus rodentium	321
DFO72_101829	Cytobacillus oceanisediminis	312
UE46_09805	Listeria weihenstephanensis	309
G7081_00870	Vagococcus coleopterorum	315
DW196_04250	Vagococcus sp. AM17-17	317
CEF21_17250	Bacillus sp. FJAT-42376	315
EBO34_10530	Bacillus sp. KQ-3	313
HMPREF9708_01310	Facklamia languida CCUG 37842	328
nrnA LCAC16_90098	Leuconostoc carnosum	323
SAMN05216231_1471	Virgibacillus salinus	315
GJR85_05935 LBAT_0464	Lactobacillus acetotolerans	318
CBF29_02165	Vagococcus elongatus	316
BN55_06070	Lactobacillus hominis DSM 23910 = CRBIP 24.179	320

EV282_1133	Fictibacillus sp. BK138	315
C269_03215	Leuconostoc gelidum (strain JB7)	338
C5L29_001833 C6Y10_13345	Lactiplantibacillus pentosus (Lactobacillus pentosus)	322
C6Y09_08480 C6Y10_14870	Lactiplantibacillus pentosus (Lactobacillus pentosus)	318
nrnA_2 JEODO184_01046	Jeotgalicoccus meleagridis	319
BUZ57_01375 GLV83_10815	Staphylococcus hyicus	311
HMPREF3106_06820	Granulicatella sp. HMSC31F03	320
DIW15_00435	Bavariicoccus seileri	321
SAMN05421687_10799	Salimicrobium flavidum	321
IV55_GL001909 LSI01_05330	Furfurilactobacillus siliginis	312
AWM72_07460 CYJ28_02295	Aerococcus sanguinicola	319
ADU72_0373 BSQ38_01590 IV84_GL000747	Pediococcus damnosus	319
SY83_16265	Paenibacillus swuensis	333
nrnA L479_00694	Exiguobacterium sp. S17	321
IV43_GL001109	Ligilactobacillus acidipiscis	321
PB1_16859	Bacillus methanolicus PB1	312
CKN67_11930 CKN75_11385	Carnobacterium divergens (Lactobacillus divergens)	318
FFL34_02845	Lentibacillus cibarius	316
BN1356_01517	Streptococcus varani	315
I532_01010	Brevibacillus borstelensis AK1	331
F9279_13785	Bacillus sp. B1-b2	310
D8M05_12555	Oceanobacillus bengalensis	317
EI998_05390	Streptococcus suis	314
FD29_GL000434	Lactobacillus mindensis DSM 14500	314
FC36_GL001153	Lactobacillus equi DSM 15833 = JCM 10991	337
FC56_GL000403	Lactobacillus senioris DSM 24302 = JCM 17472	318
FC27_GL001067	Lactobacillus versmoldensis DSM 14857 = KCTC 3814	318
nrnA SLU01_05310	Sporosarcina luteola	313
IV57_GL002049	Companilactobacillus kimchiensis	315
STRDD13_00608	Streptococcus sp. DD13	316
SINU_13300	Sporolactobacillus inulinus CASD	309
SAMN04488558_101328	Ignavigranum ruoffiae	323
RZ74_04920	Apilactobacillus apinorum	325
AZE41_07670	Sporosarcina psychrophila (Bacillus psychrophilus)	311
STRDD12_00062	Streptococcus sp. DD12	312
DS031_01640	Bacillus taeanensis	313
EWH91_01000	Sporolactobacillus sp. THM19-2	311
E1N00_03440 E1N04_06820 E1N05_10235 E1N07_08155	Staphylococcus epidermidis	312
STRIC_0055	Streptococcus ictaluri 707-05	312
OB2181	Oceanobacillus iheyensis (strain DSM 14371 / CIP 107618 / JCM 11309 / KCTC 3954 / HTE831)	315
AVJ22_07425	Staphylococcus equorum	311
HMPREF9967_0952	Streptococcus infantis SK1076	312

EQ812_09280	Staphylococcus lugdunensis	314
SAMN05216216_10763	Salinicoccus qingdaonensis	323
EV146_107141	Mesobacillus foraminis	310
NIAS840_01572	Lactobacillus salivarius NIAS840	324
SAMN05216389_111139	Oceanobacillus limi	312
STRMA_0358	Streptococcus macacae NCTC 11558	310
EDD69_10360	Thermolongibacillus altinsuensis	316
FD06_GL001161	Apilactobacillus ozensis DSM 23829 = JCM 17196	323
BZG32_07135 DAI13_06960 EU507_01600 EY666_03050 GTI81_00585 H7D67_01090 H9Q64_03670 KUB3007_C11170	Enterococcus faecalis (Streptococcus faecalis)	317
FC15_GL001480	Lactobacillus concavus DSM 17758	320
KR50_29890	Jeotgalibacillus campisalis	314
nrnA NBRC113063_00223	Apilactobacillus micheneri	326
DUD34_01155	Lactobacillus sp.	315
BUY46_09630	Staphylococcus devriesei	312
A8C40_00970	Ligilactobacillus salivarius	309
CHI06_12605	Bacillus sp. 7884-1	314
N288_19680	Bacillus infantis NRRL B-14911	310
ELX58_04820	Acetilactobacillus jinshanensis	321
EJF36_15350	Bacillus sp. HMF5848	312
SAMN04488123_10613	Natribacillus halophilus	319
nrnA_2 D8821_03640	Streptococcus gordonii	314
B808_1228	Lactobacillus florum 8D	319
Bcell_3233	Bacillus cellulosilyticus (strain ATCC 21833 / DSM 2522 / FERM P-1141 / JCM 9156 / N-4) (Evansella cellulosilytica)	313
nrnA NCTC7595_01317	Aerococcus viridans	320
SAG0136_01655	Streptococcus agalactiae LMG 14747	315
yheB Hs20B_06000	Lactococcus insecticola	311
CP356_02365	Lactobacillus sp. UMNPBX5	317
FC82_GL002695	Lactobacillus collinoides DSM 20515 = JCM 1123	312
BN822_00488	Bacillus sp. CAG:988	324
GB993_01825	Furfurilactobacillus rossiae	318
CFK37_12890	Virgibacillus phasianinus	315
A4V07_01245	Lactobacillus reuteri	319
CAT7_02257	Carnobacterium sp. AT7	316
HMPREF1210_00164	Paenisporosarcina sp. HGH0030	311
DL07_09245	Streptococcus salivarius	314
ABM34_04040	Companilactobacillus ginsenosidimutans	311
NC01_09420	Streptococcus uberis	311
C5Z25_02800	Lactobacillus sp. CBA3605	318
LRHMDP2_1193	Lactobacillus rhamnosus LRHMDP2	310
CFK40_08275	Virgibacillus necropolis	314
E4U01_03305	Streptococcus acidominimus	312

CYJ27_03855	Aerococcus christensenii	319
CBF37_09670	Vagococcus vulneris	315
C8K50_101104	Bacillus sp. YR335	312
SAMN04488100_11051	Alkalibacterium putridalgicola	321
nrnA1 FIU87_16010	Bacillus sp. THAF10	314
A499_10694	Bacillus nealsonii AAU1	310
nrnA BBEV_0880	Bacillus beveridgei	314
DES38_11412	Streptohalobacillus salinus	318
G7058_10780	Jeotgalibaca porci	320
BCR25_01990	Enterococcus termitis	316
SAMN05428946_1788	Edaphobacillus lindanitolerans	312
BFC23_07380	Carnobacterium maltaromaticum (Carnobacterium piscicola)	318
EH197_09715 GT583_09775	Enterococcus avium (Streptococcus avium)	318
D8B48_05160	Granulicatella sp.	323
HMPREF9698_00024	Alloiococcus otitis ATCC 51267	322
BU607_03250 BU608_00405	Staphylococcus auricularis	311
CYJ36_01435	Bacillus sp. UMB0893	311
FD02_GL000401	Lactobacillus nasuensis JCM 17158	319
G4D61_14210	Weizmannia ginsengihum	311
SY111_12730	Ligilactobacillus agilis	325
AL503_009290	Staphylococcus haemolyticus	312
E2K98_11295	Bacillus salipaludis	313
SAMN02746068_00668	Lactococcus chungangensis CAU 28 = DSM 22330	311
SAMN05216431_10439	Ligilactobacillus ruminis	314
WDC_1221	Paucilactobacillus wasatchensis	318
AZF04_05325	Alkalihalobacillus trypoxylicola	312
FC54_GL000417	Lactobacillus saerimneri DSM 16049	319
ER639_05955	Macrococcus sp. DPC7161	313
DFR59_103331	Falsibacillus pallidus	312
WN59_02685	Salinicoccus sediminis	289
rpmE2 E0F66_01355 G3M97_06455 GT967_03085 GT971_02875 GTK54_02450 GUA39_02600 SAMEA1711644_01780 SPNIH34_13900 SPNIH35_12150	Streptococcus pyogenes	313
LM010_05145	Lacticaseibacillus manihotivorans	312
Ssal_00913	Streptococcus salivarius (strain 57.I)	314
E4T79_01140	Streptococcus sp. LYSM12	312
HMPREF2987_01715	Streptococcus sp. HMSC067H01	311
HMPREF9088_1856	Enterococcus italicus (strain DSM 15952 / CCUG 50447 / LMG 22039 / TP 1.5)	335
B5E88_04500 EB18_01288	Enterococcus cecorum	319
CVD28_15105	Bacillus sp. M6-12	314
nrnA_1 nrnA_2 ERS132374_00895 ERS132410_00747 ERS132411_00400 ERS132444_00179	Streptococcus suis	314
AXY_09230	Amphibacillus xylanus (strain ATCC 51415 / DSM 6626 / JCM 7361 / LMG 17667 / NBRC 15112 / Ep01)	313

BKP35_12725	Anaerobacillus arseniciselenatis	316
nrnA SAMEA2297795_00184	Staphylococcus caeli	311
CIL03_00910	Virgibacillus indicus	315
AF333_10250 BN1090_A2_04457 SAMN04487909_103279	Aneurinibacillus migulanus (Bacillus migulanus)	332
HMPREF1015_00527	Bacillus smithii 7_3_47FAA	311
DFR56_11175	Pseudogracilibacillus auburnensis	314
C7384_10583	Convivina intestini	331
nrnA L248_1736	Lactobacillus shenzhenensis LY-73	320
DLJ48_00710	Oenococcus sicerae	319
nrnA_2 E4T73_08830 NCTC12413_01222	Staphylococcus arlettae	311
SAMN05216225_100521	Ornithinibacillus halophilus	316
SAMN05444126_11814	Salisediminibacterium haloalkalitolerans	313
nrnA LSA_09030	Fructilactobacillus sanfranciscensis (strain TMW 1.1304) (Lactobacillus sanfranciscensis)	319
UF66_0524	Staphylococcus cohnii subsp. cohnii	311
BH747_05235	Enterococcus villorum	316
nrnA BJR09_06580 E2557_04090 NCTC13830_01375	Staphylococcus petrasii	312
HAL01_17320	Halolactibacillus alkaliphilus	313
nrnA BN000_04413	Neobacillus massiliamazoniensis	311
SAMN05421758_10228	Salimicrobium salexigens	315
IV80_GL001390	Pediococcus cellicola	309
E2491_17170	Jeotgalibacillus sp. R-1-5s-1	310
nrnA_2 SAMEA4412661_00944	Staphylococcus muscae	311
AWH49_08130	Domibacillus aminovorans	311
LP475_02750	Limosilactobacillus pontis	318
BCR26_11325	Enterococcus rivorum	318
A374_09443	Fictibacillus macauensis ZFHKF-1	318
CF160_11690	Enterococcus pseudoavium	315
N784_01905	Pontibacillus litoralis JSM 072002	318
DI617_07210	Streptococcus pyogenes	311
IQ10_01672	Alkalihalobacillus nanhaiisediminis	311
HMPREF0501_01004	Lactobacillus coleohominis 101-4-CHN	315
GMA11_05685	Granulicatella sp. zg-ZJ	313
SAMN05216362_12435	Piscibacillus halophilus	316
D3H64_05585	Atopobacter sp. AH10	320
RU93_GL000537	Enterococcus aquimarinus	316
UB32_09910	Mesobacillus subterraneus	311
JG29_04610	Bombilactobacillus mellis	315
HMPREF1348_01493	Enterococcus faecium 505	320
JCM9157_1165	Bacillus akibai (strain ATCC 43226 / DSM 21942 / JCM 9157 / 1139)	310
AOX59_06860	Lentibacillus amyloliquefaciens	315
nrnA ytqI BSU29250	Bacillus subtilis (strain 168)	313
SAMN05421668_12328	Halolactibacillus miurensis	317

SAMN05421743_10577	Thalassobacillus cyri	320
ERX37_01025	Macrococcus hajekii	313
FKZ59_08675	Ureibacillus terrenus	314
DDV21_007800 DDV23_03680	Streptococcus chenjunshii	311
EAF07_06310	Streptococcus sp. 28462	310
FAJ39_00385 FAJ39_04205	Streptococcus suis	312
E4T78_02890	Staphylococcus lentus	308
FD21_GL000617	Lactobacillus vini DSM 20605	319
D920_02995	Enterococcus faecalis 13-SD-W-01	321
FD40_GL000869	Amylolactobacillus amylophilus DSM 20533 = JCM 1125	324
ABE65_015560	Fictibacillus phosphorivorans	315
STRDD11_02423	Streptococcus sp. DD11	323
C0213_01910	Latilactobacillus sakei (Lactobacillus sakei)	317
D1831_07310	Lactiplantibacillus garii	317
AC622_04050	Bacillus sp. FJAT-27916	313
CBF35_09285	Vagococcus salmoninarum	312
nrnA BT1A1_2473	Caldibacillus thermoamylovorans	317
FB550_104204	Neobacillus bataviensis	314
C683_1144	Catellicoccus marimammalium M35/04/3	316
X953_08660	Virgibacillus sp. SK37	313
DAT561_0988	Melissococcus plutonius	317
B1B01_00750 BEH_21210 CJ485_18085 SAMN06296056_101152	Priestia filamentosa	311
AWH56_014225 AWH56_11120	Anaerobacillus isosaccharinicus	314
SCA_1309	Staphylococcus carnosus (strain TM300)	313
EFRG_01855	Enterococcus faecium 1,231,501	320
SAMN04488137_2470	Fictibacillus solisalsi	316
AMD01_07940	Priestia koreensis	310
CHH61_06725	Alkalihalobacillus clausii (Bacillus clausii)	309
nrnA NCTC11807_01328	Staphylococcus saccharolyticus	312
C2R26_07725	Companilactobacillus formosensis	310
C273_01155	Staphylococcus massiliensis S46	310
CSE16_15985	Solibacillus sp. R5-41	313
CS060_04935	Anoxybacillus flavithermus	315
SAMN05421677_10175	Halobacillus aidingensis	319
DFR63_0485	Jeotgalicoccus halotolerans	318
SAMN04487752_2530	Carnobacterium viridans	317
EKG37_15090	Bacillus yapensis	313
SAMN05216352_102409	Alteribacillus bidgolensis	317
ESZ47_08970	Leuconostoc litchii	326
IV87_GL000344	Pediococcus ethanolidurans	316
GIW82_00805	Planomicrobium sp. YIM 101495	313
EEX84_00375	Planococcus salinus	312

nrnA_1 NCTC13765_00580	Streptococcus massiliensis	313
CR203_12810	Salipaludibacillus neizhouensis	313
CEY16_05935	Halalkalibacillus sediminis	316
NtB2_00386	Lactococcus termiticola	308
FD04_GL002099	Lactobacillus odoratitofui DSM 19909 = JCM 15043	322
DA798_04435	Lactobacillus sp. PFC-70	319
yheB Hs30E_06450	Lactococcus hodotermopsidis	311
A5867_003043	Enterococcus sp. 6D12_DIV0197	317
ESZ54_03330	Vagococcus silagei	313
GIG92_00395 GIH49_12890	Listeria monocytogenes	311
BBD26_1369	Lactobacillus delbrueckii subsp. bulgaricus	319
CBF28_08075	Vagococcus carniphilus	315
SAMN02982927_01697	Sporolactobacillus nakayamae	308
CON36_36865	Bacillus cereus	313
HMPREF9211_0426	Lactobacillus iners LactinV 01V1-a	318
CD33_04900	Lysinibacillus sinduriensis BLB-1 = JCM 15800	315
SAMN04488506_0700	Desemzia incerta	318
SAMN04490247_2717	Salimicrobium halophilum	315
nrnA NCTC12090_00787	Streptococcus equi subsp. zooepidemicus	310
BZZ03_06915	Lactococcus petauri	309
nrnA ASO14_881	Kurthia sp. 11kri321	312
FC19_GL001258	Lactobacillus aquaticus DSM 21051	320
ADA01nite_10960	Aneurinibacillus danicus	331
HMPREF3187_01465	Aerococcus christensenii	319
CQS04_00460	Chryseomicrobium excrementi	308
CR205_01030	Bacillus lacisalsi	317
CSV76_02770	Sporosarcina sp. P17b	313
BW731_03620	Vagococcus martis	317
E4U82_03515	Lentibacillus salicampi	315
FA707_09925	Vagococcus zengguangii	312
BTJ66_01125	Staphylococcus edaphicus	311
F7731_04525	Cytobacillus depressus	311
F5ESL0259_05905	Lactobacillus sp. ESL0259	318
AFK71_20910	Virgibacillus pantothenticus	314
SAMN05518872_102596	Psychrobacillus sp. OK032	309
SAMN04488134_11453	Amphibacillus marinus	317
FCL54_02100	Alkalihalobacillus caeni	313
FHL05_06810 FHL06_02420	Companilactobacillus halodurans	316
BXT97_09180 C3745_09370 GTH50_02795	Lactobacillus gasseri	321
ASG66_06240	Bacillus sp. Leaf406	312
Cdeb_03111	Caldibacillus debilis GB1	313
BCAMP_05329	Brochothrix campestris FSL F6-1037	309

FC63_GL000483	Lactobacillus amylovorus DSM 20531	318
E4T71_02450	Streptococcus sp. WM07	313
EI200_07850	Peribacillus simplex	312
F0161_08215	Paucilactobacillus nenjiangensis	316
KP78_36540	Jeotgalibacillus soli	312
DFR62_3095	Planococcus citreus	313
ELX58_06410	Acetilactobacillus jinshanensis	318
KP77_02130	Jeotgalibacillus alimentarius	311
SUB0636	Streptococcus uberis (strain ATCC BAA-854 / 0140J)	311
BAA00_06355 GT23_1905	Parageobacillus thermoglucosidasius (Geobacillus thermoglucosidasius)	315
IV83_GL001579	Pediococcus inopinatus	318
A6K76_00610	Caryophanon latum	316
CYJ72_04900	Globicatella sanguinis	323
IV56_GL001765	Lactobacillus saniviri JCM 17471 = DSM 24301	318
HM131_08135	Halobacillus mangrovi	317
WS74_0401	Weissella ceti	314
nrnA LYSIN_00119	Lysinibacillus sphaericus (Bacillus sphaericus)	315
DKZ56_11970	Ureibacillus thermophilus	314
EQJ87_03570	Lactococcus sp. S-13	312
HMPREF9706_00439	Facklamia hominis CCUG 36813	325
GLW05_11030	Pontibacillus yanchengensis	317
nrnA BN1002_03076	Bacillus sp. B-jedd	312
ADIAL_2246	Alkalibacterium sp. AK22	329
FC66_GL001518	Lactobacillus algidus DSM 15638	317
N780_02210	Pontibacillus chungwhensis BH030062	317
A7K95_10030	Pediococcus parvulus	313
SAMN04489868_1227	Pisciglobus halotolerans	326
SAMN05421737_101280	Alkalihalobacillus lonarensis	309
A6M13_00425	Caryophanon tenue	317
BD698_0439	Viridibacillus sp. OK051	313
JCM9152_1500	Bacillus hemicellulosilyticus JCM 9152	309
FC75_GL000235	Lactobacillus camelliae DSM 22697 = JCM 13995	316
NY10_2407	Carnobacterium sp. CP1	317
LB003_15240	Loigolactobacillus bifermentans	315
G4D63_09070	Bacillus mesophilus	312
FD09_GL000006	Lactobacillus perolens DSM 12744	312
nrnA DK41_03160 NCTC6175_01649 NCTC8181_00363 NCTC8185_00310 WA04_04435	Streptococcus agalactiae	311
ACA30_11220	Virgibacillus soli	310
LG542_07425	Latilactobacillus graminis	316
nrnA_1 ERS132427_01064	Streptococcus suis	314
SAMN05216244_4086	Sediminibacillus halophilus	315
IV41_GL001254	Limosilactobacillus ingluviei	320

FXE12_08865	Lactobacillus sp. SL9-6	321
GA0061094_1451	Bacillus enclensis	312
CXF70_04585	Planomicrobium sp. MB-3u-38	314
SS7213T_08077	Staphylococcus simiae CCM 7213 = CCUG 51256	313
Plano_1100	Planococcus sp. PAMC 21323	314
FEZ33_01740	Facklamia tabacinasalis	326
SAMN05192534_10451	Alteribacillus persepolensis	322
BG262_01780	Floricoccus penangensis	312
AU377_01040	Sporosarcina sp. HYO08	313
DB362_05315	Ligilactobacillus salivarius	324
nrnA JNUCC1_03208	Lentibacillus sp. JNUCC-1	315
BS756_13800	Staphylococcus sp. MB371	308
AKA01nite_10810	Alkalibacterium kapii	323
DEX24_04550	Kurthia sibirica	313
C6P08_04595 C6P11_04665	Weissella confusa (Lactobacillus confusus)	316
JCM19038_3538	Geomicrobium sp. JCM 19038	316
IV55_GL001832 LSI01_03180	Furfurilactobacillus siliginis	315
nrnA_2 BN990_01618	Virgibacillus massiliensis	315
L965_440	Leuconostoc pseudomesenteroides PS12	326
nrnA_1 SSLFYP27_02028	Staphylococcus simulans	312
DVB69_12695	Sporosarcina sp. BI001-red	313
SAMN05216243_3141	Sediminibacillus albus	315
nrnA NCTC4824_01386	Lederbergia lentus (Bacillus lentus)	310
CIL05_07915	Virgibacillus profundi	315
nrnA_2 BN997_04443	Oceanobacillus oncorhynchi	314
WOSG25_021140	Weissella oryzae (strain DSM 25784 / JCM 18191 / LMG 30913 / SG25)	314
D3H55_18110	Bacillus salacetis	312
A8F94_05815	Bacillus sp. FJAT-27225	313
AN963_23815	Brevibacillus choshinensis	331
DCM90_09075	Levilactobacillus bambusae	319
IV67_GL000745	Weissella minor	316
AT575_02135	Streptococcus penaeicida	310
MFLO_04040	Listeria floridensis FSL S10-1187	315
ADM98_14085	Exiguobacterium sp. BMC-KP	311
JCM19039_2727	Geomicrobium sp. JCM 19039	319
LRC_14020	Lactobacillus ruminis (strain ATCC 27782 / RF3)	318
nrnA BW727_100592	Jeotgalibaca dankookensis	319
FZC66_00775	Bacillus megaterium	310
GJU84_05400	Staphylococcus chromogenes (Staphylococcus hyicus subsp. chromogenes)	311
SAMN05660453_0644	Fructobacillus durionis	320
CUU64_19525	Bacillus sp. V5-8f	311
FTX54_06540	Alkalicoccus halolimnae	313

GFC30_3062	Anoxybacillus amylolyticus	315
CWO92_14450	Bacillus camelliae	317
LAV01_04490	Ligilactobacillus aviarius	322
A7K95_01205	Pediococcus parvulus	320
nrnA LPICM02_220068	Lactococcus piscium	311
FD02_GL000551	Lactobacillus nasuensis JCM 17158	310
RT41_GL000164	Lactococcus fujiensis JCM 16395	313
EWI07_06375	Sporolactobacillus sp. THM7-4	314
CQU01_17480	Cerasibacillus quisquiliarum	317
PECL_721	Pediococcus claussenii (strain ATCC BAA-344 / DSM 14800 / JCM 18046 / KCTC 3811 / P06)	317
F1591_09755	Staphylococcus sp. GDX7P459A	312
N781_09945	Pontibacillus halophilus JSM 076056 = DSM 19796	314
DCE17_04460 DCR08_01580	Lactobacillus sp.	316
NT03LS_1865	Listeria seeligeri FSL N1-067	311
HMPREF3264_03995	Staphylococcus sp. HMSC62A08	312
D1B17_03180	Companilactobacillus zhachilii	318
nrnA BTBSAS_10194	Brochothrix thermosphacta (Microbacterium thermosphactum)	310
CFN03_00975	Salinicoccus roseus	320
BTR22_02485	Alkalihalobacillus pseudofirmus (Bacillus pseudofirmus)	317
JP39_11420	Companilactobacillus heilongjiangensis	310
GWK91_08610	Virgibacillus sp. MSP4-1	321
HMPREF9421_1571	Streptococcus australis ATCC 700641	312
F7984_15215	Pradoshia sp. D12	315
FG051_00390	Companilactobacillus futsaii	310
BC6307_16885	Bacillus cohnii	312
D3873_07995	Paenisporosarcina sp. K2R23-3	313
BN609_00258	Staphylococcus sp. CAG:324	318
UC3_01753	Enterococcus phoeniculicola ATCC BAA-412	316
GZH82_06450	Staphylococcus sp. MI 10-1553	310
Q75_11540	Bacillus coahuilensis p1.1.43	316
D8M06_16905	Oceanobacillus halophilus	314
SAMN05878391_0765	Salinicoccus kekensis	323
E2556_03515	Staphylococcus croceilyticus	312
M1GAS476_0601	Streptococcus pyogenes M1 476	313
nrnA B857_03073	Solibacillus isronensis B3W22	314
nrnA C7M46_00378 GBO79_06905 GBP32_07035 IV86_GL001033	Pediococcus pentosaceus	311
nrnA EQ839_04415 H0G69_02440 LM011_05850 LMUP508_01250	Limosilactobacillus mucosae (Lactobacillus mucosae)	314
CD32_15435	Lysinibacillus odysseyi 34hs-1 = NBRC 100172	314
SAMN05192532_10325	Alteribacillus iranensis	317
FGL80_00600	Leuconostoc lactis	318
Lpp225_2528	Lactobacillus paracasei subsp. paracasei Lpp225	318
CW357_08055	Rummeliibacillus sp. TYF005	313

FG384_10170	Psychrobacillus vulpis	311
CVV77_12510	Bacillus sp. SN1	313
PL11_009835	Lentilactobacillus curieae	315
ATY39_09110	Rummeliibacillus stabekisii	311
SAMN04489735_1001223	Aneurinibacillus thermoaerophilus	332
WVI01_14040	Weissella viridescens (Lactobacillus viridescens)	332
IV88_GL000305	Pediococcus argentinicus	318
SAMN04487984_0124	Aerococcus suis	311
LPA07_10560	Lactiplantibacillus paraplantarum	318
CBF30_05465	Vagococcus entomophilus	314
CBF32_08685 DFR54_108106 HED35_00940	Vagococcus fluvialis	318
IV73_GL000822	Weissella kandleri	315
EGT49_05980	Companilactobacillus suantsaicola	313
ATL39_1454	Sinobaca qinghaiensis	319
AN959_18790	Psychrobacillus sp. FJAT-21963	309
CUU66_03745	Peribacillus deserti	314
LAC1533_1513	Ligilactobacillus acidipiscis	321
C6I21_15450	Alkalicoccus urumqiensis	311
EKG35_11670	Lysinibacillus telephonicus	314
FDZ14_27830	Bacillus megaterium	314
HMPREF9389_1808	Streptococcus sanguinis SK355	311
BAOM_4100	Peribacillus asahii	312
CHR53_21290	Neobacillus mesonae	310
SAMN05421734_10361	Pelagirhabdus alkalitolerans	311
G7082_03420	Vagococcus hydrophili	319
SAMN06295926_10743	Lysinibacillus sp. AC-3	314
FC24_GL001952	Lactobacillus rennini DSM 20253	317
UC7_01644	Enterococcus caccae ATCC BAA-1240	316
B4102_1570	Bacillus sporothermodurans	311
FD22_GL000882	Lactobacillus coryniformis subsp. coryniformis KCTC 3167 = DSM 20001	317
pde2 GAY51_07150 GC242_10865 GC243_10130 GO782_11755	Staphylococcus aureus	313
CBF27_08645	Vagococcus acidifermentans	319
RU87_GL000460	Lactococcus plantarum	311
C4B60_06105	Jeotgalibacillus proteolyticus	311
HF960_08795	Weissella hellenica	316
CUS89_00050	Enterococcus mundtii	316
FD50_GL001486	Lactobacillus satsumensis DSM 16230 = JCM 12392	319
CWS20_01985	Cytobacillus horneckiae	311
SAMN05421791_102327	Facklamia miroungae	327
E4665_05615	Sporolactobacillus shoreae	310
BHF71_00620	Vulcanibacillus modesticaldus	335
GB993_10820	Furfurilactobacillus rossiae	313

UAU_01103	Enterococcus pallens ATCC BAA-351	314
MHA01_10380	Marinococcus halophilus	319
UF70_1262	Staphylococcus pasteuri	312
SAMN05421736_116117	Evansella caseinilytica	312
LfDm3_0797	Fructilactobacillus fructivorans	318
SAMN05216179_1671	Gracilibacillus kekensis	311
BTA37_05255	Bacillus megaterium	312
EWH99_01660	Sporolactobacillus sp. THM7-7	310
E3U55_01195	Filobacillus milosensis	314
DS745_19650	Anaerobacillus alkaliphilus	314
nrnA SAMEA4412692_00145	Streptococcus merionis	310
G4D64_07765 H1Z61_08150	Bacillus aquiflavi	312
FHL04_05675	Companilactobacillus salsicarnum	318
IV87_GL000732	Pediococcus ethanolidurans	321
NRIC_12150	Enterococcus florum	316
LQ50_25550	Alkalihalobacillus okhensis	310
EV207_10154	Scopulibacillus darangshiensis	311
G7057_10135	Jeotgalibaca arthritidis	319
A9C19_04700	Bacillus weihaiensis	312
SAMN02745249_01399	Atopostipes suicloacalis DSM 15692	327
EV213_10877	Aureibacillus halotolerans	310
BKP45_11795 BKP45_17510	Anaerobacillus alkalidiazotrophicus	314
AWM75_02485	Aerococcus urinaehominis	314
ASG99_23590	Bacillus sp. Soil768D1	313
D479_07592	Halobacillus sp. BAB-2008	319
DY78_GL000592	Lactobacillus fabifermentans DSM 21115	314
nrnA_1 D8798_04330	Streptococcus cristatus	311
CD30_07865	Lysinibacillus massiliensis 4400831 = CIP 108448 = CCUG 49529	314
AM592_10635	Bacillus gobiensis	312
CHH80_05300	Bacillus sp. 7504-2	316
C6Y45_04190	Alkalicoccus saliphilus	310
CU633_09030	Bacillus sp. V3-13	311
FGL85_10425	Leuconostoc pseudomesenteroides	326
AN957_20830	Cytobacillus solani	311
UR08_04415	Listeria kieliensis	315
FC81_GL000320	Lactobacillus capillatus DSM 19910	323
BN146_09860	Lactobacillus equicursoris 66c	319
GPZ88_01310	Streptococcus sp. CNU G2	313
nrnA_1 NCTC4822_02266	Sporosarcina pasteurii (Bacillus pasteurii)	311
D0463_06955	Bacillus sp. V59.32b	310
TR210_2266	Trichococcus ilyis	320
SAMN05216187_101319	Jeotgalicoccus aerolatus	318

C7P63_04700	Vagococcus humatus	315
D8M03_00405	Lysinibacillus endophyticus	314
HMPREF3237_01920	Streptococcus sp. HMSC34B10	312
nrnA NCTC12224_01204	Streptococcus hyointestinalis	308
EDC24_0232	Aquisalibacillus elongatus	316
SKL01_02260	Staphylococcus kloosii	312
TEHN7121_1951	Tetragenococcus halophilus subsp. halophilus	318
FRX54_01035	Streptococcus sp. sy004	307
D9X91_00750	Falsibacillus albus	312
ACX53_01690 AYR53_07390	Loigolactobacillus backii	313
COD11_01700	Bacillus sp. AFS040349	312
GF867_02785 GIY09_03055 GIY11_01175	Fundicoccus ignavus	323
ESM34_05790	Staphylococcus sp. SNAZ 59	311
STO1_011990	Streptococcus oralis subsp. tigurinus	311
EQ804_05730	Staphylococcus hominis	312
BUZ14_08520	Staphylococcus gallinarum	311
CR194_11375	Salipaludibacillus keqinensis	312
BUY42_06325	Staphylococcus devriesei	312
GMB86_06485	Terrilactibacillus tamarindi	308
DZB84_08050	Bacillus sp. HNG	324
CGZ90_09930	Fictibacillus aquaticus	315
SAMN05421781_2173	Marinococcus luteus	319
E2636_05840	Paenisporosarcina antarctica	311
BFG57_03680	Bacillus solimangrovi	315
IV38_GL002102 IV40_GL001348	Lactobacillus selangorensis	318
SAMN05518684_11889	Salipaludibacillus aurantiacus	310
IV46_GL001758	Limosilactobacillus fermentum (Lactobacillus fermentum)	344
F6H94_03330	Lactobacillus jensenii	319
CBP76_03025	Companilactobacillus nuruki	312
EDM52_02930	Brevibacillus invocatus	331
HMPREF1557_01072	Streptococcus sobrinus W1703	314
nrnA_2 ERS132370_01845	Streptococcus suis	314
C5L32_000238	Lactobacillus buchneri DSM 20057	315
BTR23_07145	Alkalihalobacillus pseudofirmus (Bacillus pseudofirmus)	312
CJ205_04230	Dolosicoccus paucivorans	330
FC40_GL000523	Lactobacillus hayakitensis DSM 18933 = JCM 14209	316
GQR93_06900	Lentilactobacillus hilgardii (Lactobacillus hilgardii)	315
FC27_GL000368	Lactobacillus versmoldensis DSM 14857 = KCTC 3814	313
nrnA_2 NCTC11045_01168	Staphylococcus capitis	313
DSM07_09430	Oenococcus sp. UCMA 16435	320
C5Z26_01225	Lactobacillus sp. CBA3606	317
SAMN04487936_10166	Halobacillus dabanensis	318

nrnA AHA02nite_16750	Alkalibacillus haloalkaliphilus	314
EQG49_10080	Weissella cryptocerci	315
FC78_GL000956	Lactobacillus paralimentarius DSM 19674	315
